# A genotype and phenotype analysis of *SMAD6* mutant patients with radioulnar synostosis

**DOI:** 10.1002/mgg3.1850

**Published:** 2021-12-24

**Authors:** Fang Shen, Yongjia Yang, Pengcheng Li, Yu Zheng, Zhenqing Luo, Yuyan Fu, Guanghui Zhu, Haibo Mei, Shanlin Chen, Yimin Zhu

**Affiliations:** ^1^ The Laboratory of Genetics and Metabolism Institute of Pediatric Medicine of Hunan Province Hunan Children’s Hospital Hengyang Medical School, University of South China Changsha China; ^2^ Department of Hand Surgery Beijing Ji Shui Tan Hospital Beijing China; ^3^ Department of orthopedics Hunan Children’s Hospital Hengyang Meical School, University of South China Changsha China; ^4^ Emergency Research Institute of Hunan Province Hunan People’s Hospital Changsha China

**Keywords:** axial skeletal malformations, penetrance, pleiotropy, polydactyly, *SMAD6* variants

## Abstract

**Background:**

*SMAD6* variants have been reported in patients with radioulnar synostosis (RUS). This study aimed to investigate the genotypes and phenotypes for a large cohort of patients with RUS having mutant *SMAD6*.

**Methods:**

Genomic DNA samples were isolated from 251 RUS sporadic patients (with their parents) and 27 RUS pedigrees. Sanger sequencing was performed for the *SMAD6* coding regions. For positive probands, co‐segregation and parental‐origin analysis of *SMAD6* variants and phenotypic re‐evaluation were performed for their family members.

**Results:**

We identified 50 RUS probands with *SMAD6* variants (13 co‐segregated with RUS in pedigrees and 37 in RUS‐sporadic patients). Based on the new and previous data, we identified *SMAD6* mutated in 16/38 RUS pedigrees and 61/393 RUS sporadic patients, respectively. Overall, 93 *SMAD6* mutant patients with RUS were identified, among which 29 patients had unilateral RUS, where the left side was more involved than the right side (left:right = 20:9). Female protective effects and non‐full penetrance were observed, in which only 6.90% mothers (vs. ~50% fathers) of *SMAD6* mutant RUS probands had RUS. Pleiotropy was observed as a re‐evaluation of *SMAD6* mutant families identified: (a) three families had axial skeletal malformations; (b) two families had polydactyly; and (c) eight families had other known malformations.

**Conclusion:**

*SMAD6* was mutated in 42.11% RUS pedigrees and 15.52% RUS sporadic patients.

The RUS patients with *SMAD6* variants exhibit both non‐full‐penetrance, variable expressivity, pleiotropy, female protective effects, and the left side is more susceptible than the right side.

## Graphical abstract

Radioulnar synostosis is a rare disorder with the genetic basis keeping unknown for several 100 of years. We recently identified SMAD6 and NOG are two disease genes for it, but the data are still limited. For using such advances in clinic, we further analyzed a large cohort of patients and provided a systemic data for SMAD6 and the disease.

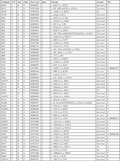

## 1 INTRODUCTION

Radioulnar synostosis (RUS, MIM: 179300) was first described by Santiford in 1793 (Simmons et al., 1983), and it is the most common congenital disorder of the elbow joint (Siemianowicz et al., 2010). In clinic, the majority of RUS are sporadic (Yang et al., 2019), in which only ~10% of RUS has family history. The inheritance of RUS is autosomal dominant (Hansen & Andersen, 1970; Rizzo et al., 1997; Spritz, 1978; Yang et al., 2019). Recently, we reported a total of 3/11 of RUS pedigrees and 24/125 of sporadic RUS patients harbored heterozygous *SMAD6* variants (Yang et al., 2019). *SMAD6* (OMIM: 602931), encodes one of the two (with *SMAD7*) inhibitory members of the SMAD family and preferentially functions in the downregulation of BMP signaling, which is essential to regulate cartilage development (Estrada et al., 2011). Interestingly, heterozygous *SMAD6* variants have also been reported on patients with congenital heart disease (CHD), bicuspid aortic valve (BAV), craniosynostosis (CS), or intellectual disability (ID; Calpena et al., 2020; Gillis et al., 2017; Jin et al., 2017; Lelieveld et al., 2016). Notably, in recessive inheritance, three *SMAD6* missense variants have been reported from two unrelated patients (one had CHD and RUS, and another had CHD only; Kloth et al., 2019).

Further, in our daily work, a previously *SMAD6*‐positive RUS family (M2553; Yang et al., 2019) consulted with our laboratory for counseling about the risk of recurrence and the possible explanations, because their new‐born baby (II:3) is suffering from CHD but without RUS and carries the same *SMAD6* variant as the family RUS proband (Figure 4).

The following points were made based on the situations above: (a) whether the mutant *SMAD6* detected in RUS can be replicated; (b) how is the transmitting features of RUS in *SMAD6* mutant families if the (a) is correct; (c) the number of *SMAD6*‐positive RUS family that co‐existed with other known *SMAD6*‐related disorder; and (d) whether the *SMAD6* mutant RUS patients exhibit other skeletal malformation given the loss of *Smad6* mice exhibiting both axial skeletal and appendicular malformations.

To answer these questions, we have further collected genomic DNA specimens from 251 RUS sporadic cases (and their family members) and 27 RUS pedigrees and performed Sanger sequencing of *SMAD6*‐coding regions for these newly collected samples. Afterward, by integrating the data (from the cases in Yang et al., 2019 and the present cases), we performed a phenotypic re‐evaluation and genotypic re‐analysis for 61 *SMAD6* mutant probands (and their family members) by focusing on RUS. We identified that *SMAD6* was mutated in 42.11% RUS pedigrees and in 15.52% RUS sporadic patients and that RUS families with *SMAD6* variants exhibited non‐full‐penetrance, variable expressivity, pleiotropy, female protective effects, and higher susceptibility at the left side than at the right side.

## 2 MATERIALS AND METHODS

### 2.1 Study subjects

Written informed consent was obtained from all probands, their parents, and their available family members. The inclusion criteria involved the diagnosis of RUS in the absence of identifiable syndromes, such as Apert/Crouzon/Pfeiffer syndrome (Schaefer et al., 1998), Holt–Oram syndrome (Wall et al., 2015), William syndrome (Charvat et al., 1991), Ehlers–Danlos Syndrome (Ritelli et al., 2017), or other obvious dysmorphic‐syndromes. None proband with amegakaryocytic thrombocytopenia or bone marrow failure (Niihori et al., 2015) were met. Patients with chromosome aneuploidy (tested by GTG banding (Yang et al., 2019) were also excluded from this study.

### 2.2 Subject classification

According to the RUS family history, we classified RUS into two categories, namely, RUS pedigree and sporadic patient. RUS pedigree indicates that a family has more than one RUS patient. RUS sporadic patient means that the family has only one RUS patient regardless of the presence of other malformations. An overall cohort description was provided in Table S3.

### 2.3 Sanger sequencing and bioinformatics analysis

For each subject, genomic DNA was extracted from peripheral blood or oral swabs by using DNA isolation kits (Cat# D3392‐02; Omega Bio‐Tek, Inc.; or Magbead Swab DNA Kit, CW2507, CoWin Biotech Co., Ltd.) in accordance with the manufacturer's procedures. Sanger sequencing was performed for the exons and intron–exon boundaries (with at least +5 and −5 bp areas were included) of *SMAD6* (NM_005585.5). Detection of 5′UTR and 3′UTR variants of *SMAD6* did not included in the present study. Polymerase chain reaction (PCR) amplification was performed using genomic DNA as a template by using a Goldstar^®^ PCR kit (Cat# CW0655M; CoWin Biotech Co., Ltd.). Sanger sequencing was conducted using a BigDye^®^ Terminator v3.1 cycle sequencing kit (Applied Biosystems, Thermo Fisher Scientific, Inc.) in accordance with the manufacturer's protocol. The amplified PCR products were purified with 70% ethanol (analytically pure) and then run on an Applied Biosystems™ 3500 series genetic analyzer (Applied Biosystems, Thermo Fisher Scientific, Inc.). Details about the primers and PCR conditions in the current study are provided in Table S2.

RUS is rare (incidence of 1/5000–10,000 in population, (Wang, 1998). Accordingly (Wang, 1998; Yang et al., 2019), only those variants that meet the following criteria remained for further evaluation: (a) rare variants (MAF < 0.0001, gnomAD_Eas or gnomAD_All); (b) variants absent in in‐house controls (479 ES data without reportable skeletal malformation); and (c) damaging variants, including loss‐of‐function variants and damaging missense variants, with damaging missense criteria of ≥2/3 in silico prediction programs, such as Mutationtaster (Schwarz et al., 2010), REVEL (Ioannidis et al., 2016), and CADD (Rentzsch et al., 2019).

### 2.4 Phenotypic investigation for families with proband having a SMAD6 variant

Considering the *SMAD6* variants enriched with CHD, BAV, CS, or ID (Calpena et al., 2020; Gillis et al., 2017; Jin et al., 2017; Kloth et al., 2019; Lelieveld et al., 2016) and *Smad6* knock‐out mice exhibiting axial skeletal malformations (Estrada et al., 2011), patients with *SMAD6*‐positive RUS probands and their available family members (those with *SMAD6* variants) were invited for counseling regarding the presence of any sign of the above disorders. Patients with possible positive signs were further invited for phenotypic re‐evaluation, which was carried out by a physician, surgeon, and geneticist, independently. When necessary, B‐ultrasound and x‐ray examinations were performed for concerned individuals.

## 3 RESULTS

### 3.1 Resequencing identified 50 *SMAD6* variants

We previously performed genetic analysis for patients with RUS (Yang et al., 2019). In the present study, we newly collected genomic DNA (with unknown cause) from 27 pedigrees and 268 sporadic cases (plus their available family members) with RUS. Sanger sequencing was firstly performed for all 295 RUS probands. We determined that all coding regions of *SMAD6* were fully covered for each (5′‐ and 3′‐UTR were not included). After filtering procedures, we identified 50 *SMAD6* rare variants (Table 1), comprising 37 loss‐of‐function, 12 damaging missense, and 1 disruptive inframe variants (Table 1).

**TABLE 1:** Phenotype–genotype list for all probands with RUS and *SMAD6* variants

Probands	P/S	Sex	Side	Position^a^	Exon	Variant	Origin^b^	Fre^c^
M3262	S	M	B	66996287	1	c.691C>T:p.R231C	Maternal	0
M3511	S	F	B	67073339	4	c.957_958insGCAA:p.A319fs	Paternal	0
M3540	S	F	B	67073842	4	c.1460G>T:p.W487L	NA	0
M1790	F	M	B	66995598	1	c.3dupG:p.M1fs	Paternal	0
R004	S	F	R	66995948	1	c.352G>T:p.E118X	Maternal	0
R005	S	M	B	67073394	4	c.1012G>T:p.E338X	Paternal	0
R016	F	F	B	66995634	1	c.38T>A:p.L13H	Paternal	0
R021	F	M	B	67004060	2	c.872delT:p.L291fs	Paternal	0
R028	S	M	B	67004047	2	c.859G>T:p.E287X	Maternal	0
R035	S	F	B	66996034	1	c.438_439insGGGGCGGCCCTGGAGCCGG:p.A146fs	Paternal	0
R041	S	M	B	67073392	4	c.1010delG:p.W337fs	Maternal	0
R052	S	F	L	66996186	1	c.590C>A:p.S197Y	Maternal	0
R073	S	F	L	67073798	4	c.1416G>A:p.W472X	Denovo	0
R074	S	M	B	66995731	1	c.135delG:p.P45fs	Paternal	0
R076	S	M	B	66996050	1	c.454_455insCGGCGGG:p.P152fs	Denovo	0
R078	S	M	B	66995822	1	c.226_250del:p.G76fs	Maternal	0
R080	F	M	B	66995598	1	c.2T>C:p.M1T	Paternal	0
R088	S	M	B	66996389	1	c.793C>T:p.H265Y	Paternal	0
R106	F	M	B	67073792	4	c.1410G>C:p.K470N	Paternal	0
R107	F	M	B	66996287	1	c.691C>A:p.R231S	Paternal	0
R108	F	M	L	67073691	4	c.1309A>T:p.K437X	Paternal	0.000004327
R118	S	M	B	66996104	1	c.508C>T:p.Q170X	Denovo	0
R026	S	M	L	66995617	1	c.21delG:p.S7fs	Maternal	0
RS021	S	M	B	66995820	1	c.224_242del:p.R75fs	Paternal	0
RJ037P1	S	M	B	66995836	1	c.240delG:p.A80fs	Maternal	0
RS014	S	M	L	66995855	1	c.259delG:p.G87fs	Maternal	0
RS139	S	M	B	66995859	1	c.264dupC:p.G88fs	Maternal	0
RS075	S	F	B	66995878	1	c.282delG:p.S94fs	NA	0
RS072	S	M	L	66995889	1	c.293delC:p.A98fs	Paternal	0
RJ027P1	S	F	L	66995920	1	c.324delG:p.A108fs	Paternal	0
RS119	S	M	B	66995661	1	c.65delG:p.R22fs	Maternal	0
RCX001	S	M	B	66995677	1	c.81_82insGGCGGCGGCGGT:p.S27delinsSGGGG	Maternal	0
RS108	S	M	B	66996387	1	c.791A>G:p.Y264C	Denovo	0
RJ050P2	F	M	B	66996389	1	c.793C>T:p.H265Y	Paternal	0
RS134	S	M	B	66996107	1	c.511G>A:p.E171K	Maternal	0
RS091	S	M	B	66996292	1	c.696G>A:p.W232X	Denovo	0
RS024	S	M	B	66996168	1	c.572T>C:p.L191P	Maternal	0.000008814
RJ002P1	S	M	B	67073606	4	c.1224delC:p.H408fs	Paternal	0
RJ004P1	S	M	B	67073685	4	c.1304dupC:p.S435fs	Maternal	0
RJ003P1	F	M	B	67073797	4	c.1415delG:p.W472fs	Paternal	0
RJ026P1	S	F	B	67073667	4	c.1285A>T:p.K429X	Maternal	0
RS033	S	M	B	67073392	4	c.1010G>A:p.W337X	Maternal	0.000004308
RJ030P1	F	M	L	67073514	4	c.1132G>T:p.E378X	Maternal	0
RS129	S	M	B	67073706	4	c.1324G>T:p.E442X	Maternal	0
RS077	S	M	B	67073377	4	c.995G>T:p.C332F	Paternal	0
M4400	S	M	B	66996185	1	c.589delT:p.S197fs	NA	0
M4553	S	F	L	66995813	1	c.217G>T:p.G73X	NA	0
M4272	S	M	R	67073480	4	c.1099dupT:p.F366fs	Paternal	0
M3996	F	M	B	66995638	1	c.42G>A:p.W14X	Maternal	0
RJ051P4	F	M	R	66995761	1	c.165C>A:p.C55X	Maternal	0

Abbreviations: B, bilateral; F, female; L, left; M, male; P, pedigrees; R, right; S, sporadic probands.

^a^ Genome position, according to Human hg19.

^b^ NA means parental genotype is unknown as DNA sample is not available.

^c^ Fre means frequency in gnomadAD_All.

### 3.2 No recessive variants on *SMAD6* were detected

For recessive *SMAD6* variants reported on complex CHD/RUS patients (Kloth et al., 2019), we checked if the RUS proband in the present study carries *SMAD6* recessive variants. However, results showed that none of the probands carried rare *SMAD6* recessive variants (Table 1). Even after using a less stringent filtering condition by adjusting MAF to less than 0.01 (gnomAD_Eas) and expanding the data by adding 24 previously reported sporadic RUS patients, further analysis (Yang et al., 2019) indicated that none of them have *SMAD6* recessive variants (data not shown).

### 3.3 Features of RUS pedigrees with *SMAD6* variants

**FIGURE 1:**
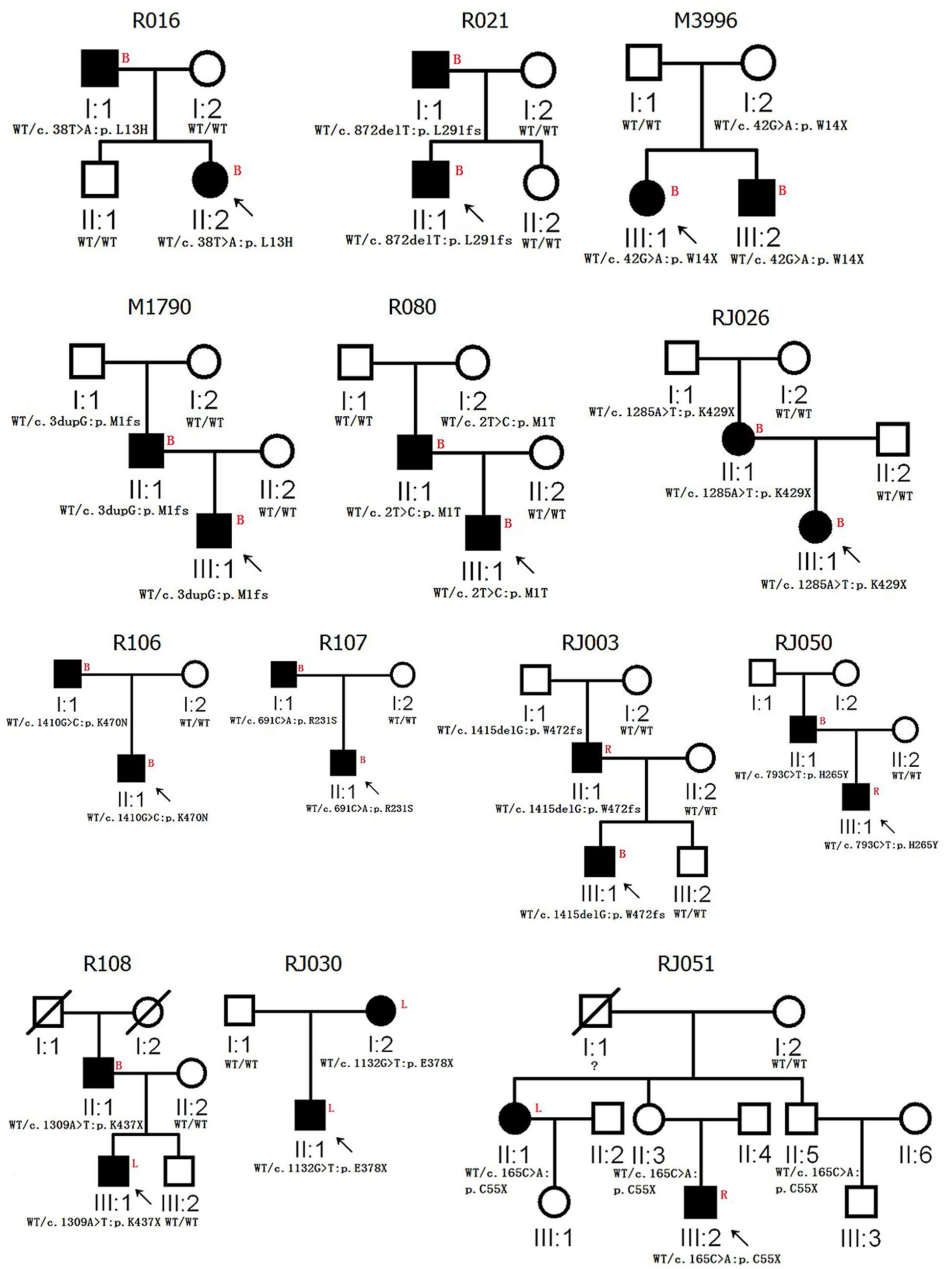
The newly identified 13 RUS pedigrees with mutant *SMAD6*. For the red alphabet, L mean Left, R mean Right, B mean Bilateral; WT mean wild type

From 27 RUS pedigrees, 13 probands carried *SMAD6* rare variants (Table 1). All 13 *SMAD6* variants co‐segregated with RUS in each of the pedigrees with non‐full penetrance were observed (Figure 1, Figure S1). In combination with our previous data in which 3/11 RUS pedigrees had *SMAD6* variants, *SMAD6* was mutated in 42.11% (16/38) RUS pedigrees. Based on these *SMAD6*‐positive pedigrees, the inheritance of RUS was autosomal dominant with 13 times of vertical transmission of RUS in these 16 pedigrees (Figure 1). These 13 vertical transmissions included 10 male‐to‐male, 1 male‐to‐female, 1 female‐to‐male, and 1 female‐to‐female transmission (Figure 1).

### 3.4 Features of RUS sporadic patients with *SMAD6* variants

A total of 37/268 RUS sporadic patients had *SMAD6* variants (Table 1, Figure S2). In combination with previous data (*SMAD6* mutant in 24/125 RUS sporadic cases) (Yang et al., 2019), *SMAD6* was mutated in 61/393 (15.52%) sporadic patients with RUS.

Among these 393 sporadic patients, 285 were males and 108 were females. In RUS males, 49/285 had *SMAD6* variants (17.19%). In RUS females, 12/108 had *SMAD6* variants (11.11%).

Herein, parental DNA samples were available for 45/61 *SMAD6* positive RUS sporadic probands. Further, Sanger sequencing on their parents identified that 9/45 (20%) are de novo (the paternity relationship for each family was validated, data not shown), 24/36 variants (66.7%) have a maternal origin, and 12/36 (33.3%) variants have a paternal origin (Table 1; Figure S2).

### 3.5 Non‐full penetrance, variable expressivity, and the Carter effect

#### 3.5.1 The penetrance was not full

By focusing on RUS, we studied 431 probands (393 sporadic and 38 probands from pedigrees). Exactly 77/431 probands were *SMAD6*‐positive (50 variants identified in here and 27 from a previous study (Yang et al., 2019)). Exactly 61/77 had parental genomic DNA. Among these variants, 9/61 variants were de novo. For the 52 remaining probands, 23 have a paternal origin. Among the 23 fathers with *SMAD6* variants, 11 had RUS (47.83%). For the 29 remaining variants with maternal origin, 2/29 *SMAD6*‐mutated mothers had RUS (6.90%).

#### 3.5.2 Expressivity was variable

At least three points can prove that *SMAD6*‐mutated RUS patients are associated with variable expressivity. First, in 61 sporadic RUS patients with *SMAD6* variants, 19 (31.15%) were unilaterally affected (Table 1, Table S3). Second, in 32 RUS patients from 16 *SMAD6* mutated pedigrees, 10 patients have unilateral RUS (31.25%, Figure 1, Table S3). Third, within a pedigree, family members with the same *SMAD6* variant can exhibit bilateral or unilateral RUS. For example, in pedigrees, R108 and RJ050, fathers (II:1) had bilateral RUS but their children (III:1) only had unilateral RUS (Figure 1). In family RJ003, father (II:1) had right RUS, but his son (III:1) had bilateral RUS (Figure 1).

The Carter effect, also known as female protective effect (Carter, 1961), was identified in *SMAD6*‐mutated RUS patients. First, the number of males was higher than that of females. As previously described, the male‐to‐female ratio of sporadic RUS was 3:1 (Yang et al., 2019). In the present study, the male‐to‐female ratio for *SMAD6*‐positive sporadic RUS patients was 4.10:1 (49 males vs. 12 females). Also, in *SMAD6*‐positive RUS pedigrees, RUS males (Jordan et al., 2012a) were more than RUS females (Estrada et al., 2011), with the male to female ratio of 3.6:1. Second, the penetrance of RUS for *SMAD6*‐positive parents (of the probands) varied (maternal: 6.90% vs. paternal: 47.83%). Third, in mutated patients, the RUS was less severe in females than that in males. RUS can be bilateral or unilateral. In the present study, we identified 93 SMAD6‐mutated RUS patients (Schwarz et al., 2010). In 72 males, 19 were unilateral (26.39%). In 21 females, 10 were unilateral (47.62%). Therefore, if we define the unilateral RUS as less severe, the females with the *SMAD6* variant tend to have less severe RUS.

### 3.6 Phenomenon of the left side were more susceptible

In 29/93 *SMAD6*‐mutated patients with unilateral RUS (Table S3), 20 were affected at the left side, while 9 were affected at the right side, indicating that the left side was more susceptible than the right side. Further, we notified that the left and right differences occurred in sporadic patients. In *SMAD6* mutant sporadic patients, 19/61 patients had unilateral RUS, in which 15/19 were affected at the right side, while 4/19 were affected at the right side. In unilateral RUS patients from *SMAD6* mutant pedigrees, 5/10 were affected at the left side, while 5/10 were affected at the right side.

### 3.7 Pleiotropy: Novel (polydactyly, spinal malformations) and known phenotypes identified in *SMAD6* mutant families

In combination with data obtained from previous cases (Yang et al., 2019), 77 *SMAD6* mutant probands with RUS were identified. We intended to recall all patients and their available relatives for clinical re‐evaluation. However, several early patients lost to follow‐up. In total, 61 *SMAD6* mutant probands (and their family members) participated in our program for the further survey about other possible phenotypes (except for RUS). A total of 13/61 families had other related malformations. This figure was possibly under‐estimated because only individuals (or family members) with identifiable symptoms underwent further clinical examination.

**FIGURE 2:**
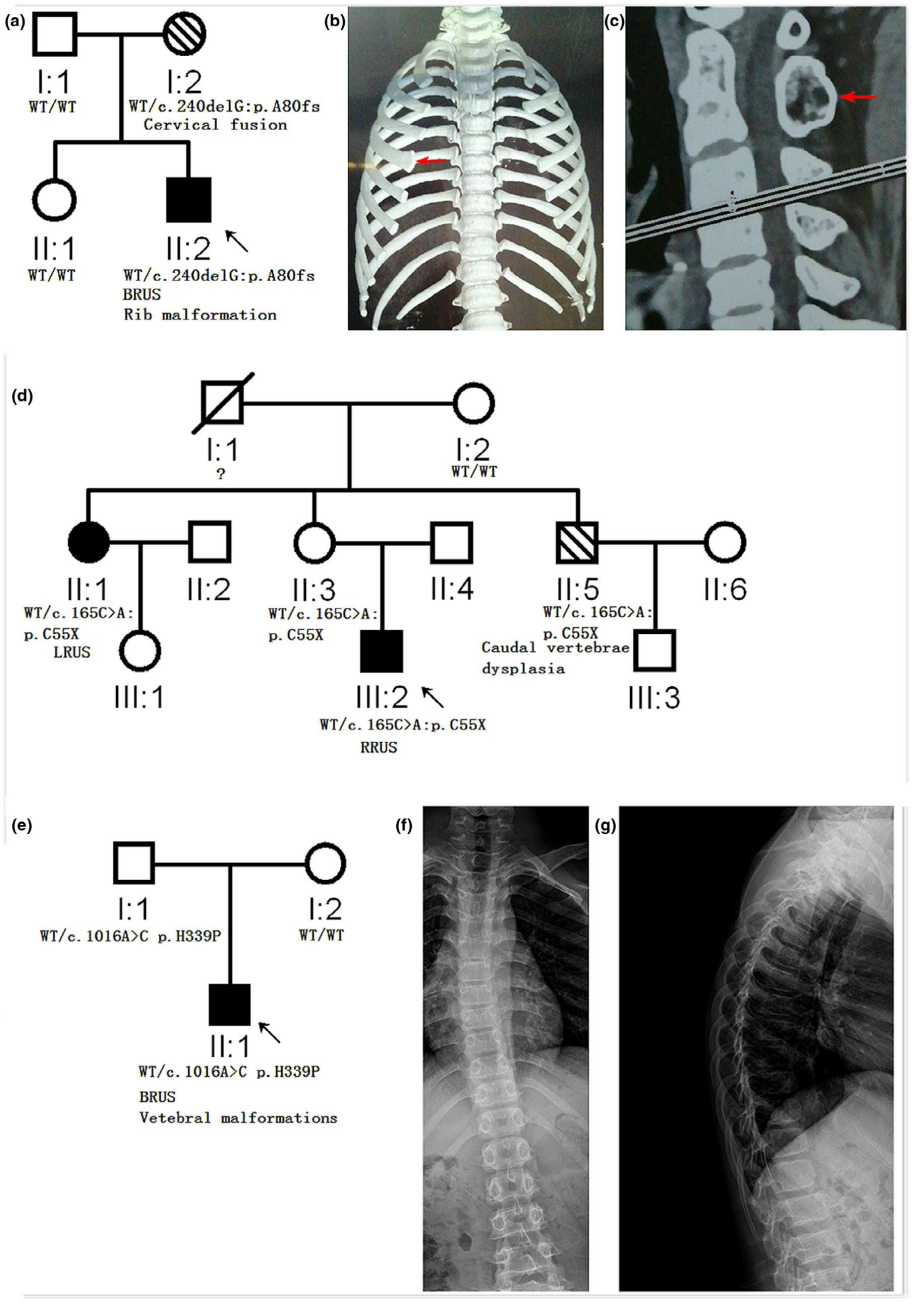
Three *SMAD6* mutant RUS families with axial skeletal malformations. (a) The family RJ037. (b) The x‐ray images of the fourth rib‐malformation (arrow) of RJ037‐II:2. (c) The CT image of the cervical vertebrae fusion (arrow) of RJ037‐I:2. (d) The family RJ051, the II:5 had vertebra malformations at young age (but develops to normal at 28 years old). (e) The x‐ray image of the caudal vertebra dysplasia and lumbar vertebra degeneration of RJ051‐II:5. (f) Family M1204, this case was reported previously (Yang et al., 2019). (g, h) x‐ray images of scoliosis (g), kyphosis, vertebral bone osteosclerosis, and microshrinkage (h) of M1204

Three families had axial skeletal malformations. The present study identified three RUS families with mutant *SMAD6* had axial skeletal deformities (Figure 2). In family RJ037 (Figure 2a), both the proband (II:2) and his mother I:2 carried the same *SMAD6* loss of function variant (c.240delG/p.A80fs), II:2 suffered from RUS and enlarged the fourth rib at the right side (Figure 2b), but his mother (I:2) suffered from bone fusion that occurred between the first and second cervical vertebrae (Figure 2c). In family RJ051 (Figure 2d), III:2, II:1, and II:5 all carried a *SMAD6* loss‐of‐function variant (c.165C>A/p.C55X), III:2, and II:1 had RUS, but II:5 had caudal vertebra dysplasia without RUS. In family M1204 (c.1016A>C/p.H339P, (Yang et al., 2019), Figure 2e), the proband suffered from RUS and spinal malformations, including scoliosis, kyphosis, vertebral bone osteosclerosis, and micro‐shrinkage (Figure 2f,g). We have also observed that another seven *SMAD6* mutant members in RUS families had kyphosis, (5/7 were less than 40‐years‐old) and six *SMAD6* mutant members in RUS families had obvious vertebral degeneration. Considering that the incidence of kyphosis or vertebral degeneration is high in the general population, we cannot define a definite association between the *SMAD6* variant and kyphosis or vertebral degeneration at present.

**FIGURE 3:**
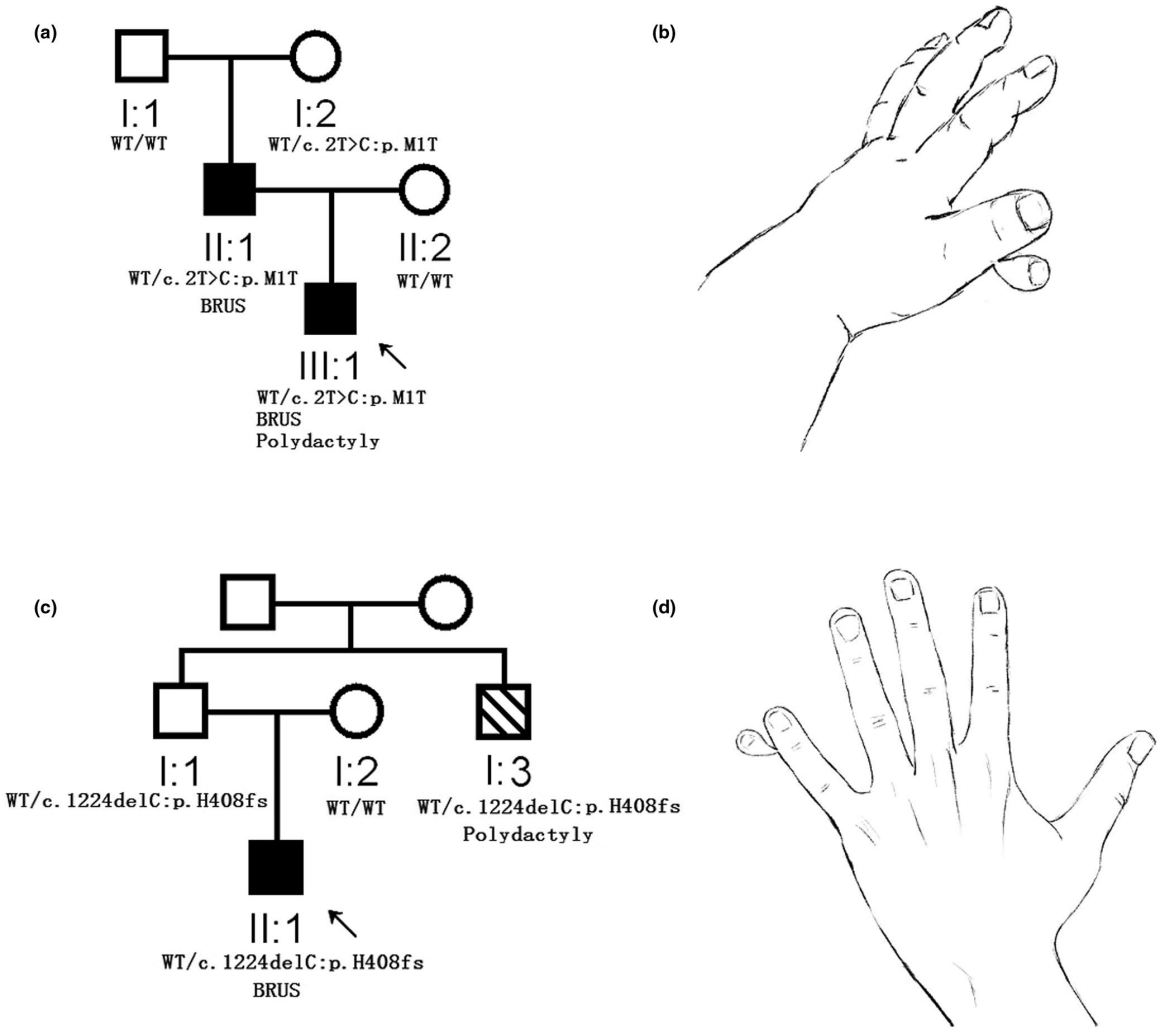
Two *SMAD6* mutant RUS families with polydactyly. (a) Family R080. (b) The polydactyly image of R080‐III:1. (c) Family RJ002. (d) The polydactyly image of RJ002‐II:3. Since both the patients with polydactyly underwent surgery at an early age, the polydactyly pictures were drawn based on the recollections of the patient's parents

Two RUS‐positive families had polydactyly. In family R080, the proband (*SMAD6*: c.2T>C/p.M1T) suffered from RUS and left thumb polydactyly (extra floating finger) (Figure 3a,b). In family RJ002, the proband II:1 suffered from RUS, but his uncle I:3 suffered from left fifth finger polydactyly (also extra floating finger, Figure 3c,d) but without RUS, and both of them had SMAD6:c.1224delC/p.H408fs.

**FIGURE 4:**
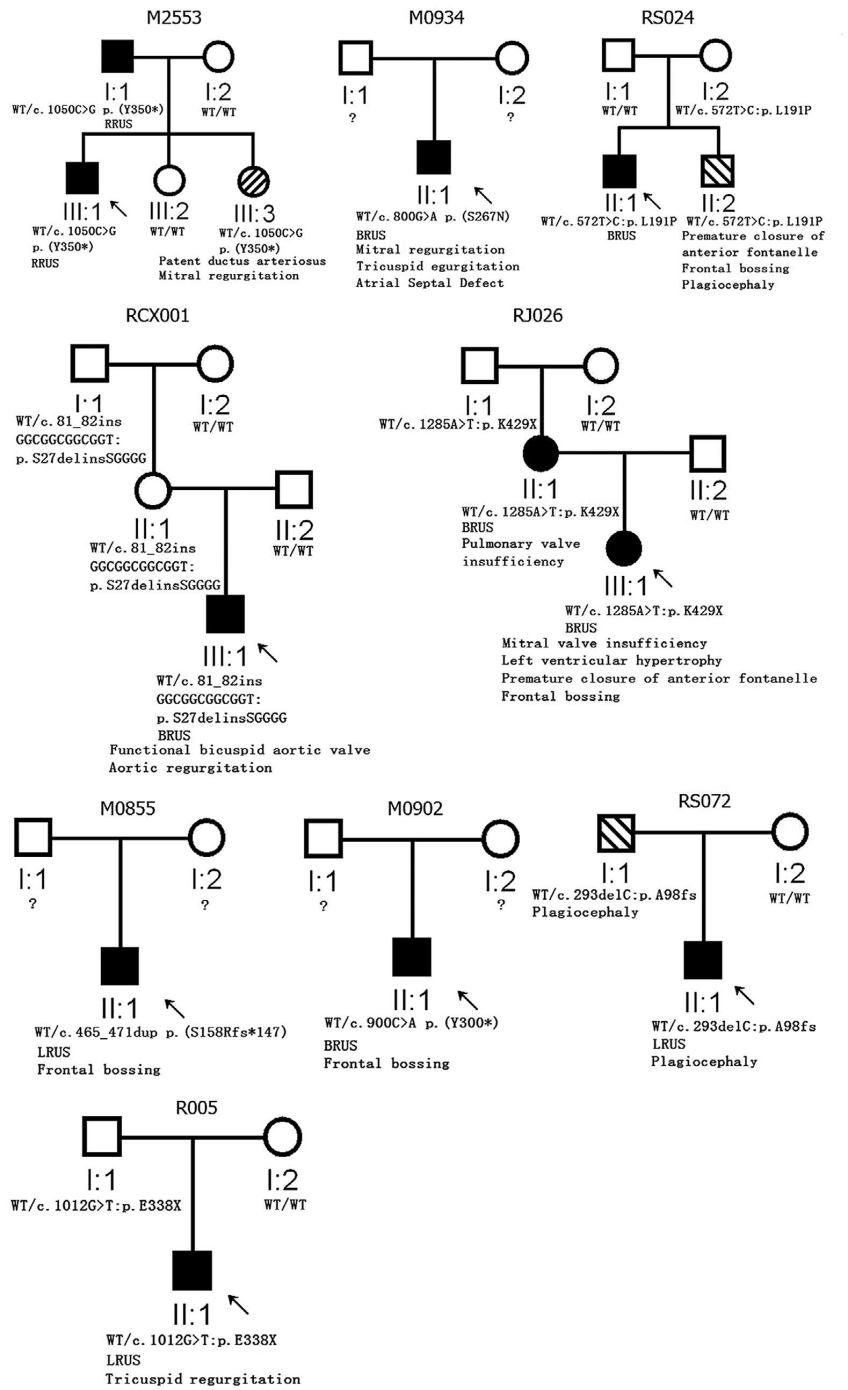
Nine *SMAD6* mutant RUS families with other known phenotypes. Genotypes (WT: wild type) and phenotypes (LRUS, RRUS, and BRUS mean Left, Right and Bilateral RUS, respectively) were illustrated under each individual

Nine RUS families had other known phenotypes. Based on the re‐evaluation, we identified 6/61 *SMAD6*‐positive RUS families (seven patients) with CHD (with one had BAV, Figure 4). Typically, in a previously reported *SMAD6* positive family M2553, I:1, III:1, and III:3 all had a *SMAD6* loss‐of‐function variant (c.1050C>G/p.Y350X). However, the I:1 and III:1 suffered from RUS, but the newly born individual III:3 suffered from CHD (patent ductus arteriosus and mild mitral regurgitation) without RUS (Figure 4). In family R005 (Figure 4), both the individuals I:1 and II:1 carried the same *SMAD6* variant (c.1012G>t/p.E338X), I:1 was normal, but his son II:1 suffered from RUS and CHD (mild tricuspid regurgitation). Similar findings for the four other *SMAD6* mutant RUS families with CHD are illustrated in Figure 4.

We observed that six *SMAD6* mutant family members suffered from skull abnormalities, in which three had frontal bossing, two had plagiocephaly, and one had both frontal bossing and plagiocephaly (note: 4/6 had both RUS and skull abnormalities, Figure 4). Notably, we did not identify any *SMAD6* rare variants from four RUS patients with intellectual disability, and none of the *SMAD6* mutant members of these 61 families has reached the point that an intelligence test is required.

## 4 DISCUSSION

By focusing on RUS, the present study identified 50 rare *SMAD6* variants through the 295 probands obtained from 268 RUS sporadic patients and 27 RUS pedigrees. We first determined that 37/50 variants were deleterious because 37 *SMAD6* variants were loss‐of‐function variants (comprising of 21 frameshift, 14 stop‐gain, 2 initiation codon variants, Table 1). These 37 variants tended to produce abnormal mRNA that is generally associated with nonsense‐mediated decay and then exerted a haploinsufficiency effect as the mechanism. Second, we determined that 12/50 *SMAD6* missense variants were all deleterious on the basis of the following considerations: (a) any of these 12 variants did not exist on genomAD databases and in our 479 in‐house exome sequencing databases; (b) Calpena et al. (2020) designed a model to evaluate in silico the deleteriousness of *SMAD6* missense variants (such model was based on functional experiments data). According to Calpena model, if a *SMAD6* variant fulfills both DS > 4 and the CADD predicted damaging, such *SMAD6* missense should be defined as deleterious. In the present study, all 12 *SMAD6* missense variants met the above criteria (Table S2) and were thus defined as deleterious. Only one *SMAD6* variant, that is, the c.81_82insGGCGGCGGCGGT:p.S27delinsSGGGG that identified from family RXC001, should be defined as uncertain significance, because this variant was not reported in the gnomadAD database or in our in‐house 479 exome database, and the proband with such variant had both RUS and BAV (both two disorders were specific to *SMAD6* disruption (Gillis et al., 2017; Yang et al., 2019). Moreover, at the same position of S27, 49 deletion alleles (15‐66995669‐GGCGGCA‐G, inframe deletion, p.Ser27_Gly28del) are reported in gnomadAD database. Further functional experiments are needed to test the pathogenicity of this variant.

From the 61 *SMAD6*‐mutated probands (who stayed in touch), we identified that 14 families had subordinate clinical findings. Notably, in the three families, *SMAD6*‐mutant members had axial skeletal malformations, comprising one patient with cervical vertebrae‐fusion, one patient with rib malformation, one patient with caudal vertebral dysplasia, and one patient with scoliosis and kyphosis. Loss of *Smad6* in mice leads to defects in both axial and appendicular skeletal development (Estrada et al., 2011). Specifically, *Smad6*−/− mice exhibited a posterior transformation of the seventh cervical vertebra, bilateral ossification centers in lumbar vertebra, and bifid sternebrae caused by incomplete sterna band fusion (Estrada et al., 2011). Therefore, the *SMAD6*‐mutated individuals who exhibit axial skeletal malformations support the skeletal phenotypes of *Smad6*−/− mice, suggesting that *SMAD6* mutant patients should focus on the presence of axial skeletal malformations. Another novel incidental phenotype on RUS families identified in the present study was polydactyly. Two *SMAD6* mutant members had polydactyly, both two additional digits were connected to the fingers just like a nubbin (Figure 3). In one of the affected hands, the extra finger was attached to the thumb (the radial side). In another hand, the extra finger was attached to the small finger (the ulnar side). Considering that the frequency of polydactyly was as rare as 1 in ~700–1000 live births (Jordan et al., 2012a), and *SMAD6* was involved in the antagonizing BMP‐signaling (such signaling disruption involved in the number of phalanges in animals (Jordan et al., 2012b)), it is unlikely that polydactyly occurred on such two *SMAD6* mutant families was a coincidence. Further confirmative experiments are needed.

From the *SMAD6*‐mutated probands, we also identified eight families in which the family members suffered from CHD, BAV, or skull abnormalities. *SMAD6* variants enriched in CHD, BAV, or skull malformation have been well described previously (Calpena et al., 2020; Gillis et al., 2017; Jin et al., 2017; Kloth et al., 2019; Lelieveld et al., 2016). The present study confirmed that different phenotypes can occur in different members (with the same mutant *SMAD6*) within a family. Therefore, CHD, BAV, RUS, skull abnormalities, axial skeletal malformation, and polydactyly should be concluded to *SMAD6*‐related phenotypic spectrum.

The Carter effect, which was observed by Cedric Carter in patients with pyloric stenosis in 1961, refers to females that are less commonly affected by pyloric stenosis and are more likely than males with pyloric stenosis to have children affected with the disorder (Carter, 1961; Carter & Evans, 1969). In the present study, we found that the Carter effect is remarkably associated with the RUS phenotype in patients with *SMAD6* variants as several obvious female protective effects observed which were described above. However, one point of the present study did not fit to Carter effect. According to the Carter effect, the affected female should carry more severe (or increased number of) variants than that of the affected male, and the relatives of the affected female have more chance to develop the index disorder. In the present study, 3/11 (27.3%) *SMAD6* mutant female probands had a family relatives suffered by RUS. By comparison, 13/50 *SMAD6* mutant males (26.0%) had family history of RUS, and we did not observe female RUS probands with mutant *SMAD6* having more family history of RUS.

In *SMAD6* mutant patients with unilateral RUS, the number of left RUS was remarkably higher than that of right RUS. *SMAD6* encodes an inhibitory component of BMP/SMAD signaling (Estrada et al., 2011). It is known the lateral plate mesoderm (LPM) forms the progenitor cells that constitute the limb skeleton, heart and cardiovascular system, and others (Prummel et al., 2020) in the developing vertebrate embryo. Considering that the BMP/SMAD signaling sets a repressive threshold in the LPM essential for the integrity of LEFT/RIGHT signaling (Furtado et al., 2008), *SMAD6* haploinsufficiency may affect the integrity of LEFT/RIGHT signaling, causing asymmetric development of the left/right limbs. Vertebrate embryo development is not a complete symmetric event because many organs (such as stomach, heart, spleen, etc.) primary located on the left side, and a more precise BMP signal is needed on the left side development. Therefore, the left side was more susceptible to developing RUS under *SMAD6* haploinsufficiency.

## CONFLICT OF INTEREST

The authors have declared no conflict of interest.

## AUTHOR CONTRIBUTIONS

Conceptualization: Yongjia Yang, Yimin Zhu, Shanlin Chen and Fang Shen. Data curation: Yongjia Yang, Yimin Zhu. Funding acquisition: Yongjia Yang, Yimin Zhu. Investigation: Fang Shen, Pengcheng Li, Yu Zheng, Yuyan Fu, Guanghui Zhu. Methodology: Haibo Mei, Yu Zheng, Yongjia Yang, Resources: Haibo Mei, Shanlin Chen, Yongjia Yang. Supervision: Yongjia Yang, Yimin Zhu. Writing—original draft: Yongjia Yang and Fang Shen. Writing—review and editing: Yongjia Yang, Yimin Zhu, Shanlin Chen.

## ETHICAL COMPLIANCE

The present study was approved by the Ethics Committee of Hunan Children's Hospital (HCHLL58, Changsha City, Hunan Province, China) and the Ethics Committee of Beijing Jishuitan Hospital (202101‐09, Beijing, China).

## Supporting information

Fig S1‐S2

Table S1‐S4

## Data Availability

The datasets generated and/or analyzed during the current study are available in the National Center for Biotechnology Information (NCBI) ClinVar repository, (https://www.ncbi.nlm.nih.gov/clinvar/variation/SUB9887746/).

## References

[mgg31850-bib-0001] Calpena, Eduardo , Cuellar, Araceli , Bala, Krithi , Swagemakers, Sigrid M. A. , Koelling, Nils , McGowan, Simon J. , Phipps, Julie M. , Balasubramanian, Meena , Cunningham, Michael L. , Douzgou, Sofia , Lattanzi, Wanda , Morton, Jenny E. V. , Shears, Deborah , Weber, Astrid , Wilson, Louise C. , Lord, Helen , Lester, Tracy , Johnson, David , Wall, Steven A. , Twigg, Stephen R. F. , Mathijssen, Irene M. J. , Boardman‐Pretty, Freya , Boyadjiev, Simeon A. , & Wilkie, Andrew O. M. (2020). SMAD6 variants in craniosynostosis: genotype and phenotype evaluation. Genetics in Medicine, 22(9), 1498–1506. 10.1038/s41436-020-0817-2 32499606 PMC7462747

[mgg31850-bib-0002] Carter, C. O. (1961). The inheritance of congenital pyloric stenosis. British Medical Bulletin, 17, 251–254. 13691133 10.1093/oxfordjournals.bmb.a069918

[mgg31850-bib-0003] Carter, C. O. , & Evans, K. A. (1969). Inheritance of congenital pyloric stenosis. Journal of Medical Genetics, 6, 233–254. 5345095 10.1136/jmg.6.3.233 PMC1468738

[mgg31850-bib-0004] Charvat, K. A. , Hornstein, L. , & Pediatr, O. A. E. (1991). Radio‐ulnar synostosis in Williams syndrome. Pediatric Radiology, 21(7), 508–510. 10.1007/BF02011725 1771116

[mgg31850-bib-0006] Estrada, K. D. , Retting, K. N. , Chin, A. M. , & Lyons, K. M. (2011). Smad6 is essential to limit BMP signaling during cartilage development. Journal of Bone and Mineral Research, 26, 2498–2510. 10.1002/jbmr.443 21681813 PMC3183270

[mgg31850-bib-0007] Furtado, M. B. , Solloway, M. J. , Jones, V. J. , Costa, M. W. , Biben, C. , Wolstein, O. , Preis, J. I. , Sparrow, D. B. , Saga, Y. , Dunwoodie, S. L. , Robertson, E. J. , Tam, P. P. L. , & Harvey, R. P. (2008). BMP/SMAD1 signaling sets a threshold for the left/right pathway in lateral plate mesoderm and limits availability of SMAD4. Genes & Development, 22(21), 3037–3049. 10.1101/gad.1682108 18981480 PMC2577791

[mgg31850-bib-0008] Gillis, Elisabeth , Kumar, Ajay A. , Luyckx, Ilse , Preuss, Christoph , Cannaerts, Elyssa , van de Beek, Gerarda , Wieschendorf, Björn , Alaerts, Maaike , Bolar, Nikhita , Vandeweyer, Geert , Meester, Josephina , Wünnemann, Florian , Gould, Russell A. , Zhurayev, Rustam , Zerbino, Dmytro , Mohamed, Salah A. , Mital, Seema , Mertens, Luc , Björck, Hanna M. , Franco‐Cereceda, Anders , McCallion, Andrew S. , Van Laer, Lut , Verhagen, Judith M. A. , van de Laar, Ingrid M. B. H. , Wessels, Marja W. , Messas, Emmanuel , Goudot, Guillaume , Nemcikova, Michaela , Krebsova, Alice , Kempers, Marlies , Salemink, Simone , Duijnhouwer, Toon , Jeunemaitre, Xavier , Albuisson, Juliette , Eriksson, Per , Andelfinger, Gregor , Dietz, Harry C. , Verstraeten, Aline , & Loeys, Bart L. (2017). Candidate gene resequencing in a large bicuspid aortic valve‐associated thoracic aortic aneurysm cohort: SMAD6 as an important contributor. Frontiers in Physiology, 8, 400. 10.3389/fphys.2017.00400 28659821 PMC5469151

[mgg31850-bib-0009] Hansen, O. H. , & Andersen, N. O. (1970). Congenital radio‐ulnar synostosis: report of 37 cases. Acta Orthopaedica Scandinavica, 41, 225–230. 5486179 10.3109/17453677008991509

[mgg31850-bib-0010] Ioannidis, N. M. , Rothstein, J. H. , Pejaver, V. , Middha, S. , McDonnell, S. K. , Baheti, S. , Musolf, A. , Li, Q. , Holzinger, E. , Karyadi, D. , Cannon‐Albright, L. A. , Teerlink, C. C. , Stanford, J. L. , Isaacs, W. B. , Xu, J. , Cooney, K. A. , Lange, E. M. , Schleutker, J. , Carpten, J. D. , … Sieh, W. (2016). REVEL: An ensemble method for predicting the pathogenicity of rare missense variants. American Journal of Human Genetics, 99(4), 877–885. 10.1016/j.ajhg.2016.08.016 27666373 PMC5065685

[mgg31850-bib-0011] Jin, Sheng Chih , Homsy, Jason , Zaidi, Samir , Lu, Qiongshi , Morton, Sarah , DePalma, Steven R. , Zeng, Xue , Qi, Hongjian , Chang, Weni , Sierant, Michael C. , Hung, Wei‐Chien , Haider, Shozeb , Zhang, Junhui , Knight, James , Bjornson, Robert D. , Castaldi, Christopher , Tikhonoa, Irina R. , Bilguvar, Kaya , Mane, Shrikant M. , Sanders, Stephan J. , Mital, Seema , Russell, Mark W. , Gaynor, J. William , Deanfield, John , Giardini, Alessandro , Porter, George A. , Srivastava, Deepak , Lo, Cecelia W. , Shen, Yufeng , Watkins, W. Scott , Yandell, Mark , Yost, H. Joseph , Tristani‐Firouzi, Martin , Newburger, Jane W. , Roberts, Amy E. , Kim, Richard , Zhao, Hongyu , Kaltman, Jonathan R. , Goldmuntz, Elizabeth , Chung, Wendy K. , Seidman, Jonathan G. , Gelb, Bruce D. , Seidman, Christine E. , Lifton, Richard P. , & Brueckner, Martina (2017). Contribution of rare inherited and de novo variants in 2,871 congenital heart disease probands. Nature Genetics, 49(11), 1593–1601. 10.1038/ng.3970 28991257 PMC5675000

[mgg31850-bib-0012] Jordan, D. , Hindocha, S. , Dhital, M. , Saleh, M. , & Khan, W. (2012). The epidemiology, genetics and future management of syndactyly. The Open Orthopaedics Journal, 6(1), 14–27. 10.2174/1874325001206010014 22448207 PMC3308320

[mgg31850-bib-0013] Jordan, D. , Hindocha, S. , Dhital, M. , Saleh, M. , & Khan, W. (2012). The epidemiology, genetics and future management of syndactyly. The Open Orthopaedics Journal, 6, 14–27. 10.2174/1874325001206010014 22448207 PMC3308320

[mgg31850-bib-0014] Spritz, R. A. (1978). Familial radioulnar synostosis. Journal of Medical Genetics, 15(2), 160–162. 10.1136/jmg.15.2.160 641954 PMC1013669

[mgg31850-bib-0015] Kloth, Katja , Bierhals, Tatjana , Johannsen, Jessika , Harms, Frederike L. , Juusola, Jane , Johnson, Mark C. , Grange, Dorothy K. , & Kutsche, Kerstin (2019). Biallelic variants in SMAD6 are associated with a complex cardiovascular phenotype. Human Genetics, 138(6), 625–634. 10.1007/s00439-019-02011-x 30963242

[mgg31850-bib-0016] Lelieveld, Stefan H. , Reijnders, Margot R. F. , Pfundt, Rolph , Yntema, Helger G. , Kamsteeg, Erik‐Jan , de Vries, Petra , de Vries, Bert B. A. , Willemsen, Marjolein H. , Kleefstra, Tjitske , Löhner, Katharina , Vreeburg, Maaike , Stevens, Servi J. C. , van der Burgt, Ineke , Bongers, Ernie M. H. F. , Stegmann, Alexander P. A. , Rump, Patrick , Rinne, Tuula , Nelen, Marcel R. , Veltman, Joris A. , Vissers, Lisenka E. L. M. , Brunner, Han G. , & Gilissen, Christian (2016). Meta‐analysis of 2,104 trios provides support for 10 new genes for intellectual disability. Nature Neuroscience, 19(9), 1194–1196. 10.1038/nn.4352 27479843

[mgg31850-bib-0017] Niihori, T. , Ouchi‐Uchiyama, M. , Sasahara, Y. , Kaneko, T. , Hashii, Y. , Irie, M. , Sato, A. , Saito‐Nanjo, Y. , Funayama, R. , Nagashima, T. , Inoue, S. , Nakayama, K. , Ozono, K. , Kure, S. , Matsubara, Y. , Imaizumi, M. , & Aoki, Y. (2015). Mutations in MECOM, encoding oncoprotein EVI1, cause radioulnar synostosis with amegakaryocytic thrombocytopenia. American Journal of Human Genetics, 97(6), 848–854. 10.1016/j.ajhg.2015.10.010 26581901 PMC4678429

[mgg31850-bib-0018] Prummel, Karin D. , Nieuwenhuize, Susan , & Mosimann, Christian (2020). The lateral plate mesoderm. Development, 147(12), 175059. 10.1242/dev.175059 PMC7328003 32561665

[mgg31850-bib-0019] Rentzsch, Philipp , Witten, Daniela , Cooper, Gregory M. , Shendure, Jay , & Kircher, Martin (2019). CADD: Predicting the deleteriousness of variants throughout the human genome. Nucleic Acids Research, 47(D1), D886–D894. 10.1093/nar/gky1016 30371827 PMC6323892

[mgg31850-bib-0020] Ritelli, M. , Dordoni, C. , Cinquina, V. , Venturini, M. , Calzavara‐Pinton, P. , & Colombi, M. (2017). Expanding the clinical and mutational spectrum of B4GALT7‐spondylodysplastic Ehlers‐Danlos syndrome. Orphanet Journal of Rare Diseases, 12(1), 153. 10.1186/s13023-017-0704-3 28882145 PMC5590203

[mgg31850-bib-0021] Rizzo, R. , Pavone, V. , Corsello, G. , Sorge, G. , Neri, G. , & Opitz, J. M. (1997). Autosomal dominant and sporadic radio‐ulnar synostosis. American Journal of Medical Genetics, 68, 127–134. 10.1002/(SICI)1096-8628(19970120)68:2<127:AID-AJMG2>3.0.CO;2-M 9028445

[mgg31850-bib-0022] Schaefer, F. , Anderson, C. , Can, B. , & Say, B. (1998). Novel mutation in the FGFR2 gene at the same codon as the Crouzon syndrome mutations in a severe Pfeiffer syndrome type 2 case. American Journal of Medical Genetics, 75(3), 252–255. 10.1002/(SICI)1096-8628(19980123)75:3<252:AID-AJMG4>3.0.CO;2-S 9475591

[mgg31850-bib-0023] Schwarz, J. M. , Rödelsperger, C. , Schuelke, M. , & Seelow, D. (2010). MutationTaster evaluates disease‐causing potential of sequence alterations. Nature Methods, 7(8), 575–576. 10.1038/nmeth0810-575 20676075

[mgg31850-bib-0024] Siemianowicz, A. , Wawrzynek, W. , & Besler, K. (2010). Congenital radioulnar synostosis – Case report. Polish Journal of Radiology, 75, 51–54. PMC3389900 22802806

[mgg31850-bib-0025] Simmons, B. P. , Southmayd, W. W. , & Riseborough, E. J. (1983). Congenital radioulnar synostosis. Journal of Hand Surgery. American, 8, 829–838. 10.1016/s0363-5023(83)80078-1 6643957

[mgg31850-bib-0026] Wall, L. B. , Piper, S. L. , Habenicht, R. , Oishi, S. N. , Ezaki, M. , & Goldfarb, C. A. (2015). Defining features of the upper extremity in holt‐oram syndrome. The Journal of Hand Surgery, 40(9), 1764–1768. 10.1016/j.jhsa.2015.06.102 26243320 PMC4757499

[mgg31850-bib-0027] Wang, J. (1998). Congenital radioulnar synostosis. In: S. Ji , S. Pan , & J. Wang (Eds), Pediatric Orthopaedics (In Chinese, pp. 92–94). Shandong Science and Technology Press.

[mgg31850-bib-0028] Yang, Y. , Zheng, Y. , Li, W. , Li, L. , Tu, M. , Zhao, L. , Mei, H. , Zhu, G. , & Zhu, Y. (2019). SMAD6 is frequently mutated in nonsyndromic radioulnar synostosis. Genetics in Medicine, 21(11), 2577–2585. 10.1038/s41436-019-0552-8 31138930

