# Supplementary material for: A genotype and phenotype analysis of SMAD6 mutant patients with radioulnar synostosis
Source: Mol Genet Genomic Med. 2021 Dec 24;10(1):e1850. doi: 10.1002/mgg3.1850 (PMC8801148; doi:10.1002/mgg3.1850)

**Supplementary Figure 1:** Sanger sequencing results of 13 RUS pedigrees with mutant *SMAD6*

Note: each page represents a separate RUS pedigree, upper panel represents the family pedigree, the lower panel represents the trace figure of the Sanger sequencing.

RJ050


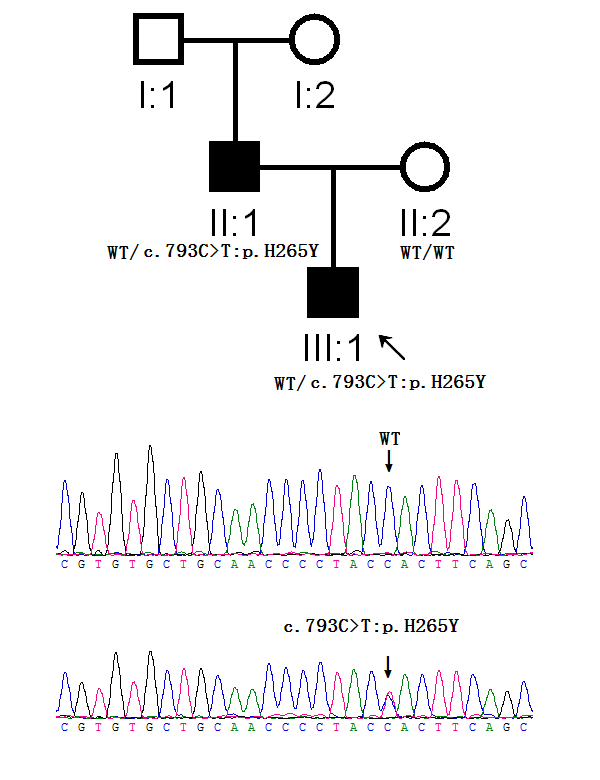


RJ030


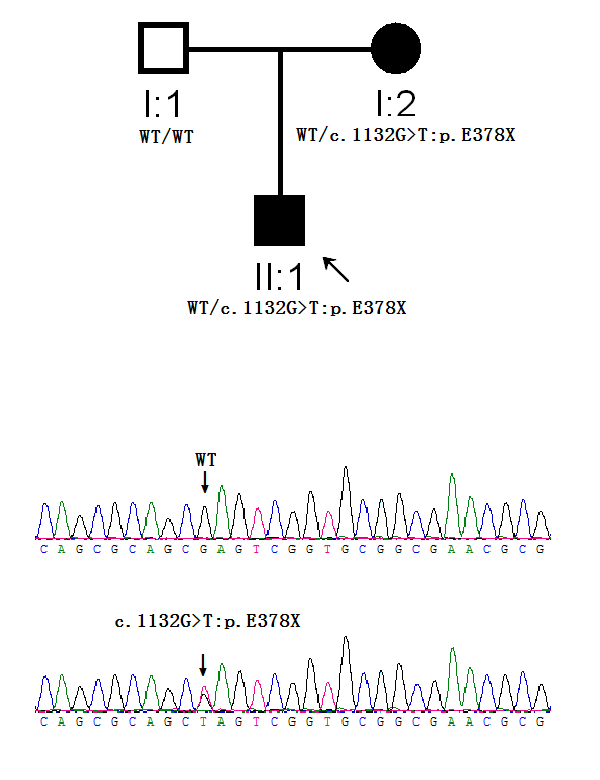


RJ026


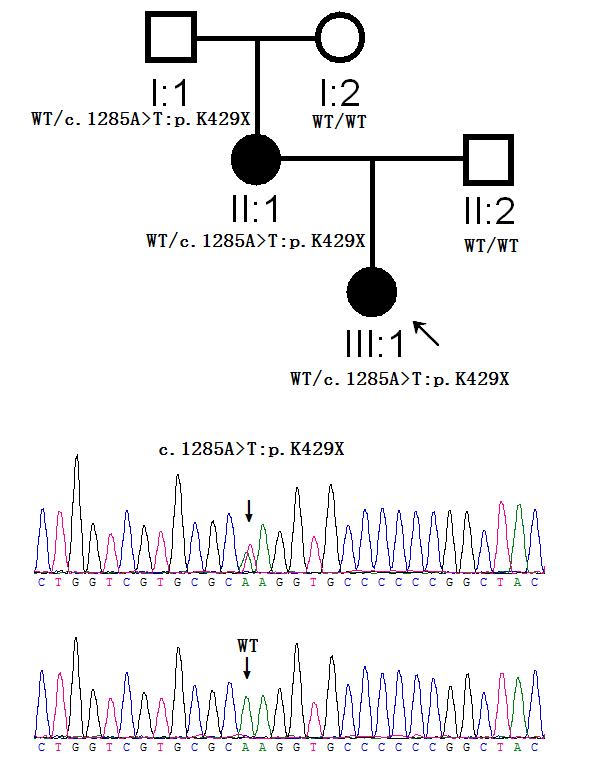


RJ003


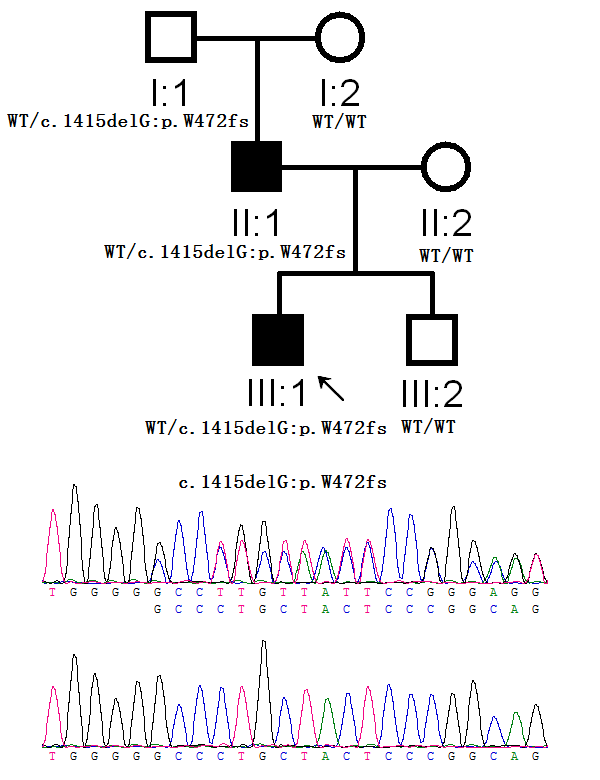


R108


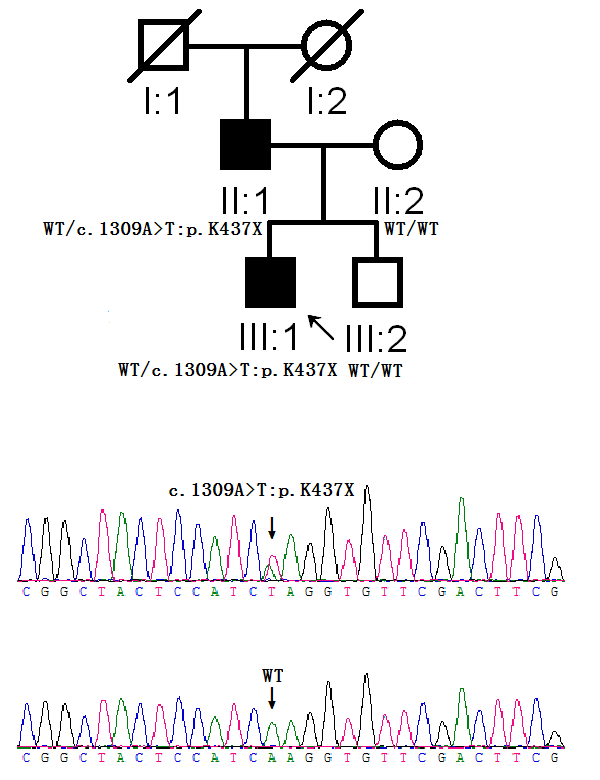


R107


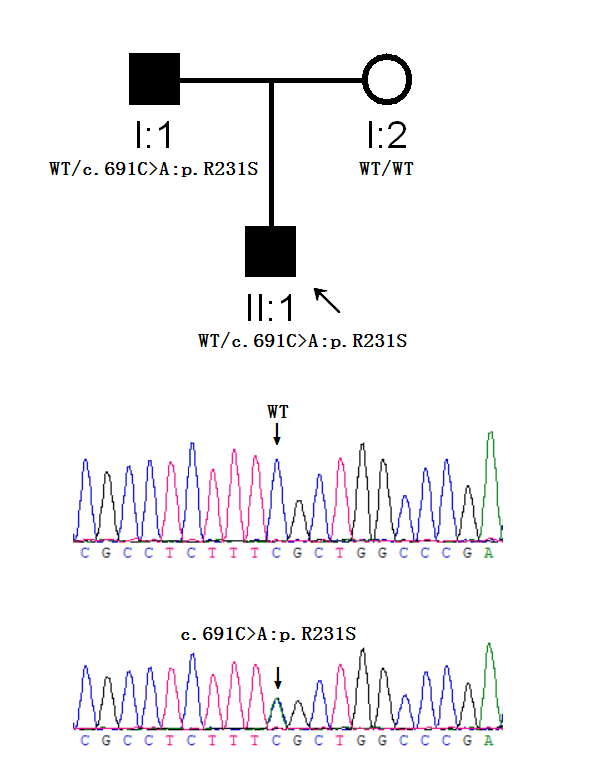


R106


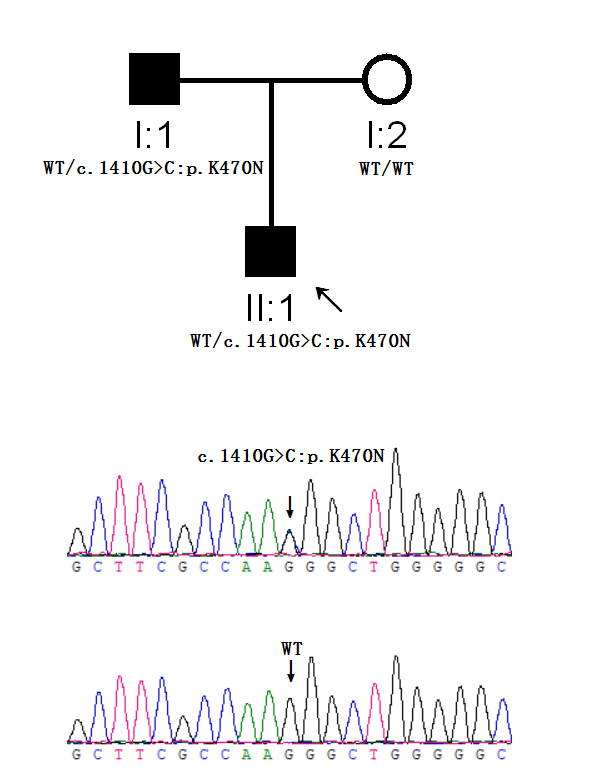


R080


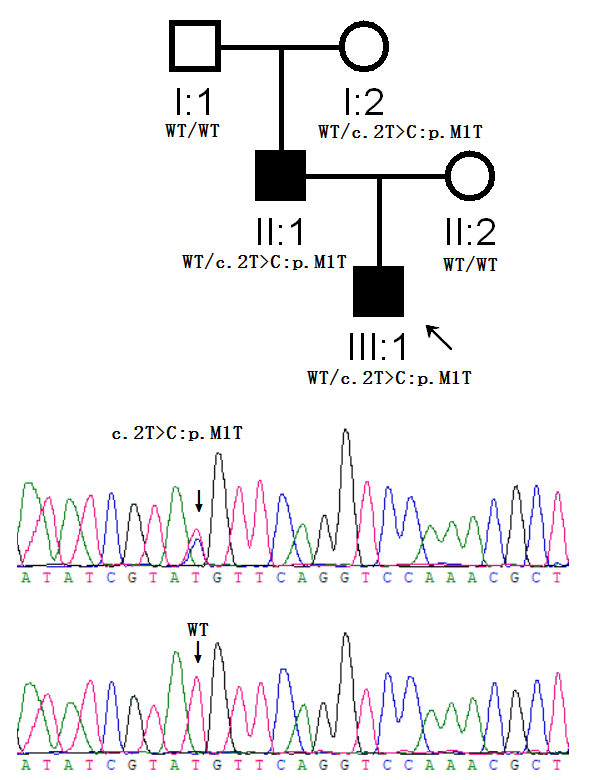


R021


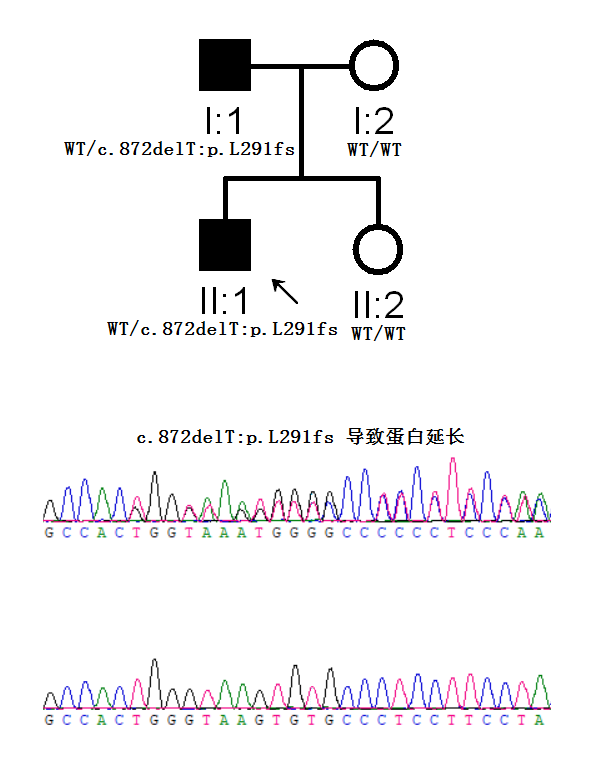


R016


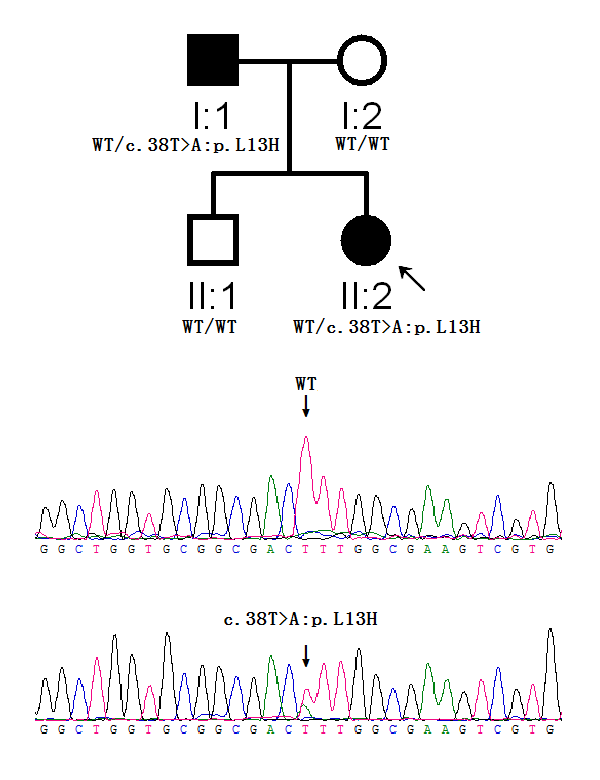


M3996


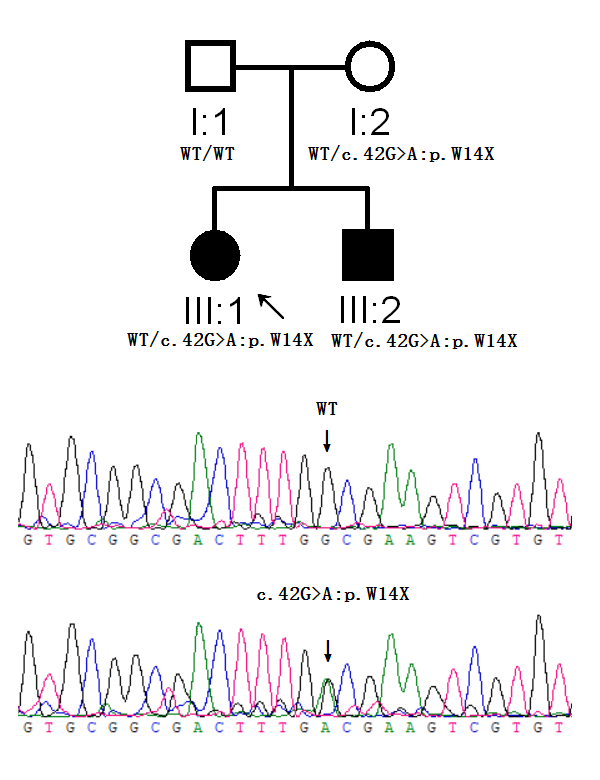


M1790


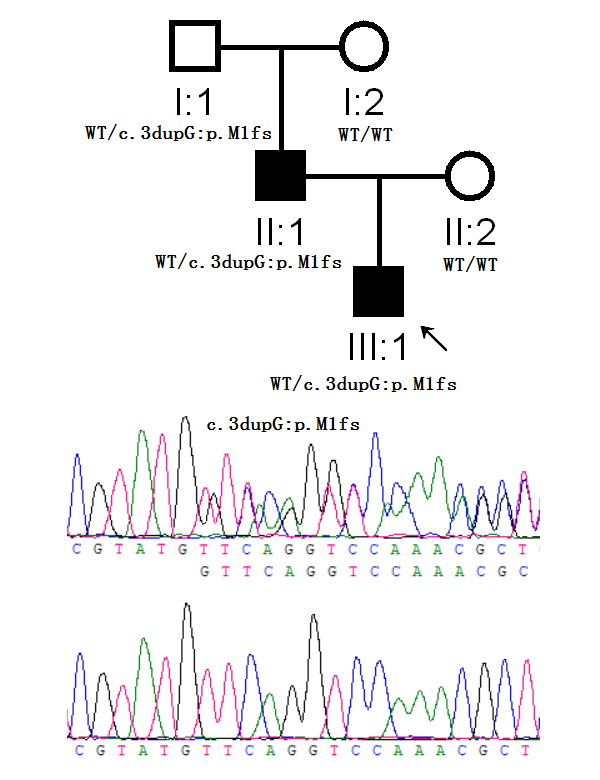


RJ051


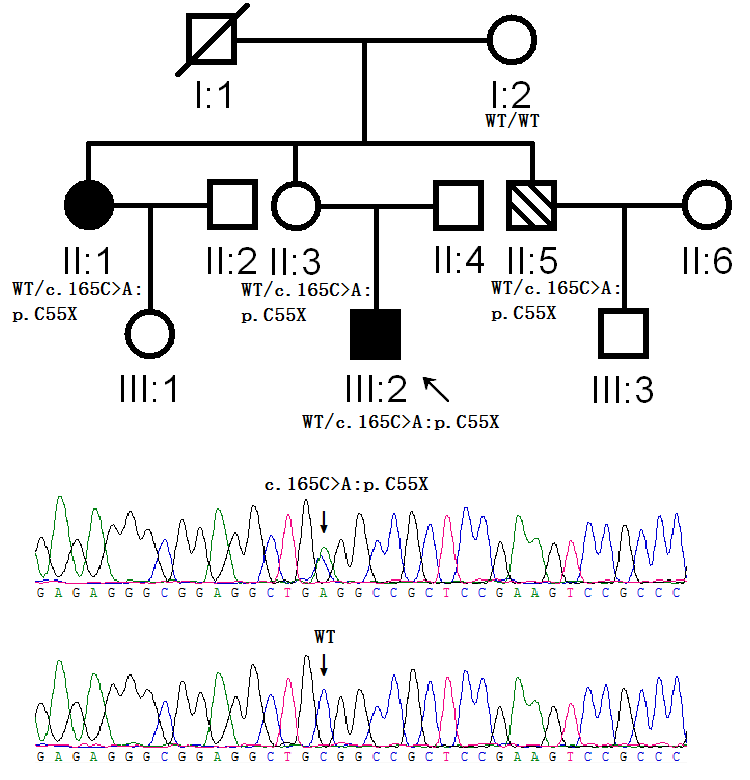


**Supplementary Figure 2:** Sanger sequencing results of 37 RUS sporadic patients with mutant *SMAD6* (genotypes for their available family members were provided). Note: each page represents a separate RUS sporadic patient, the upper panel represents the family, the lower panel represents the trace figure of the Sanger sequencing.

RS134
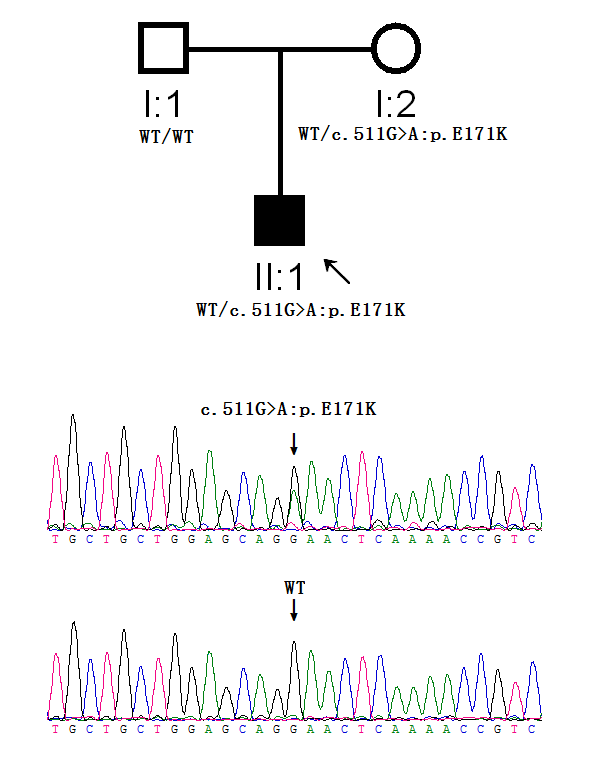


RS129
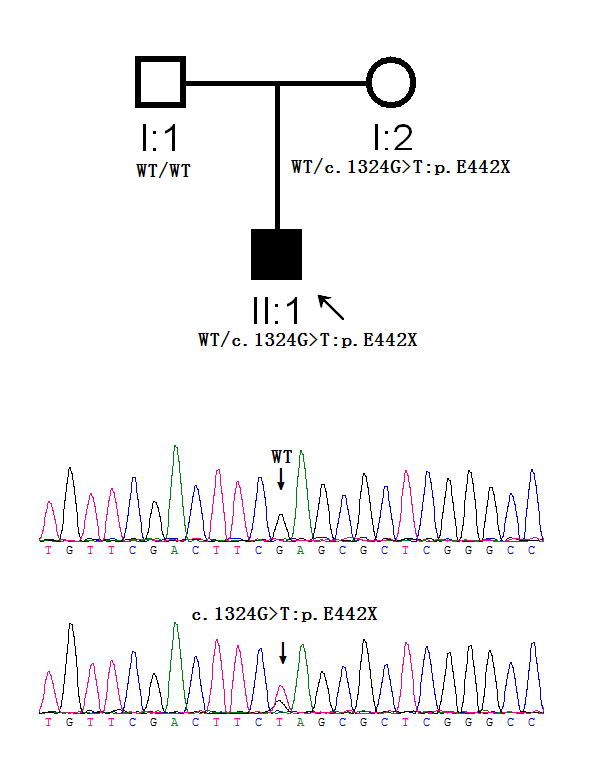


RS119
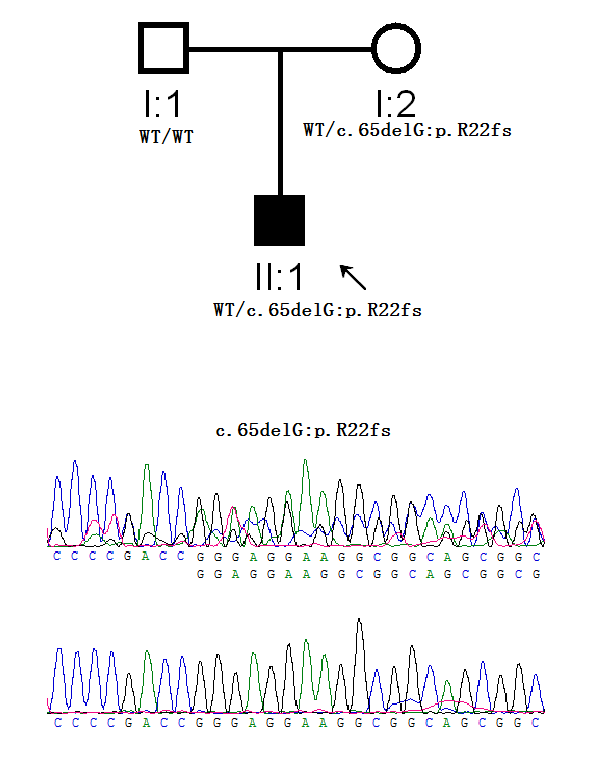


RS108
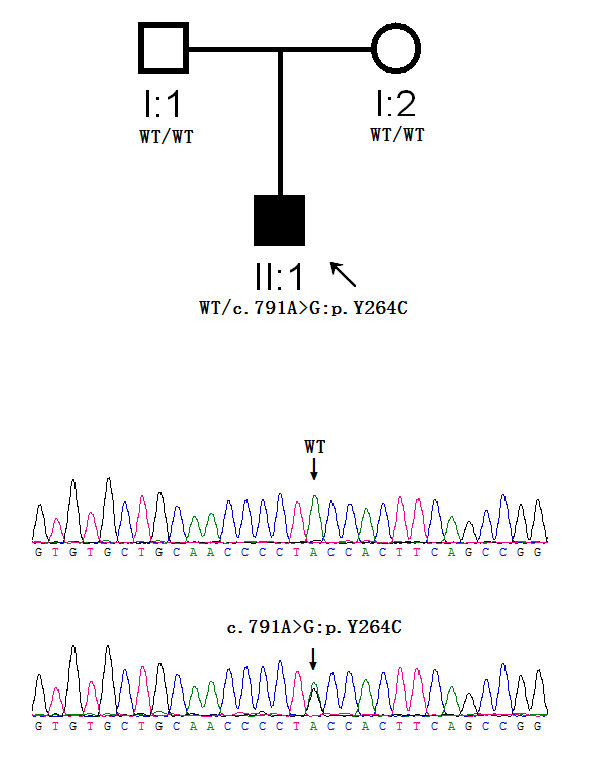


RS091
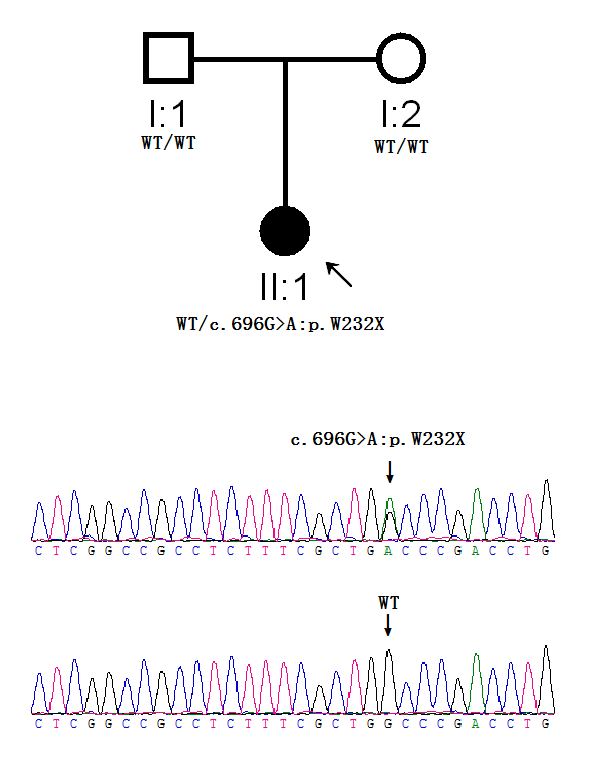


RS077
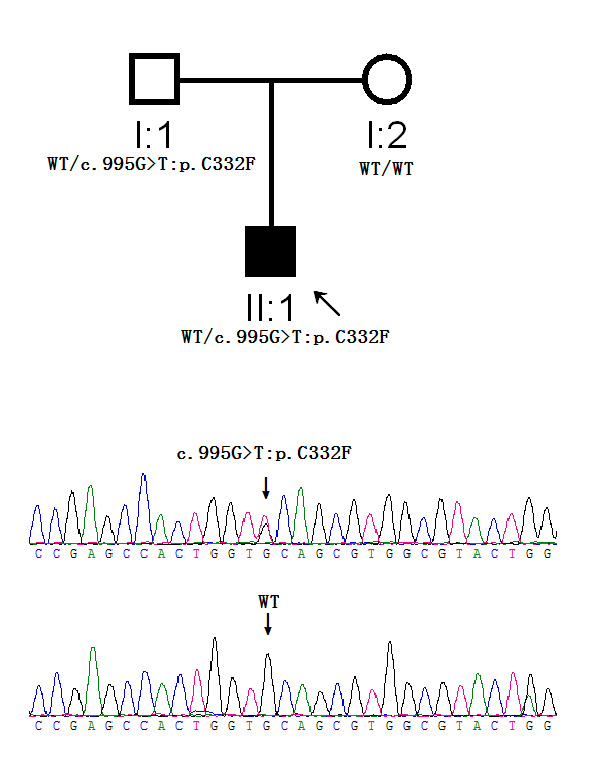


RS075
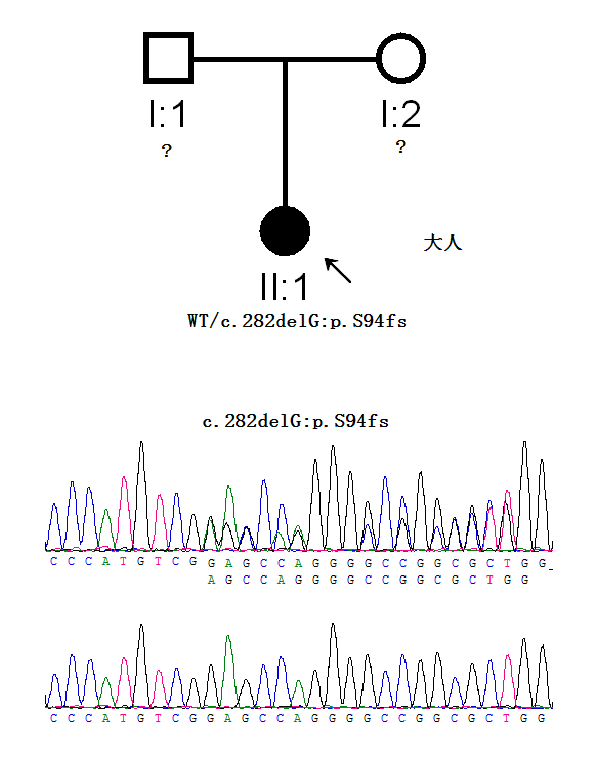


RS072
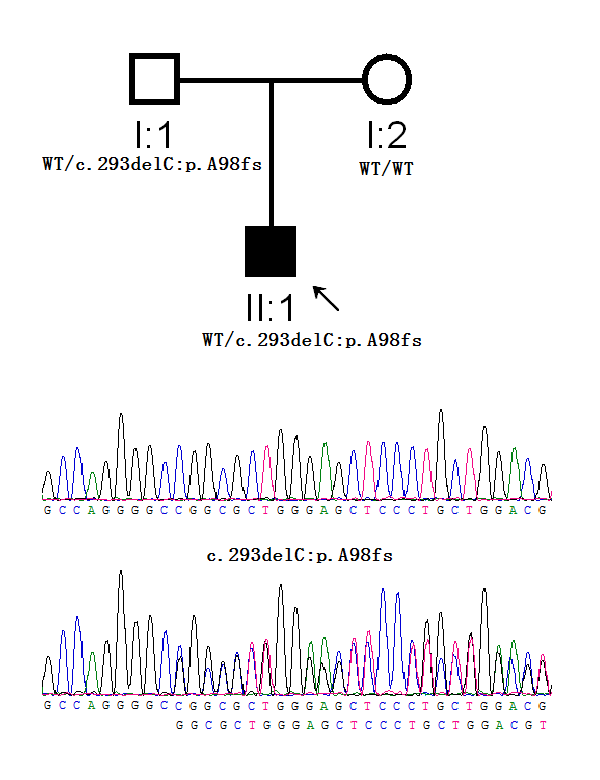


RS033
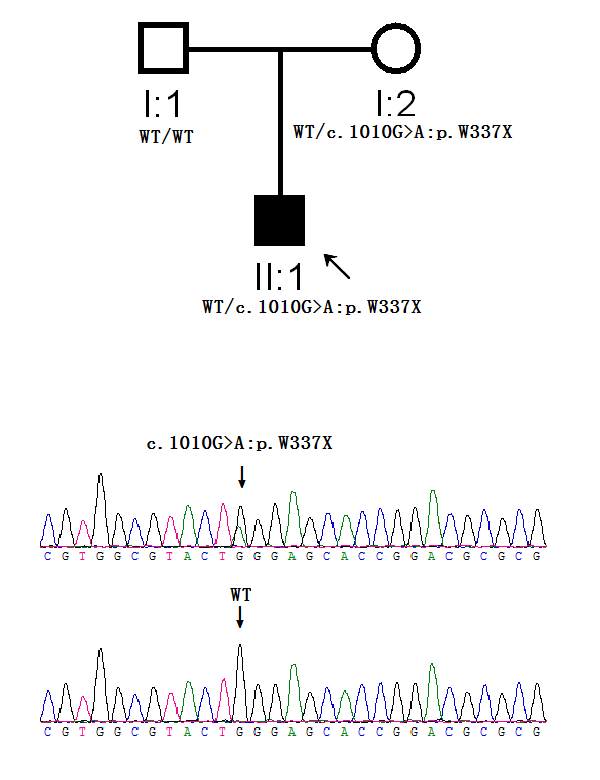


RS024
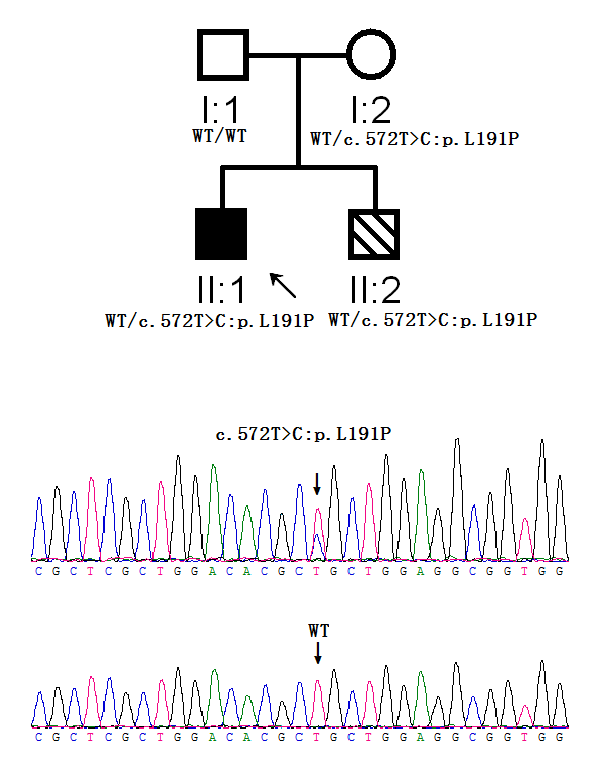


RS021
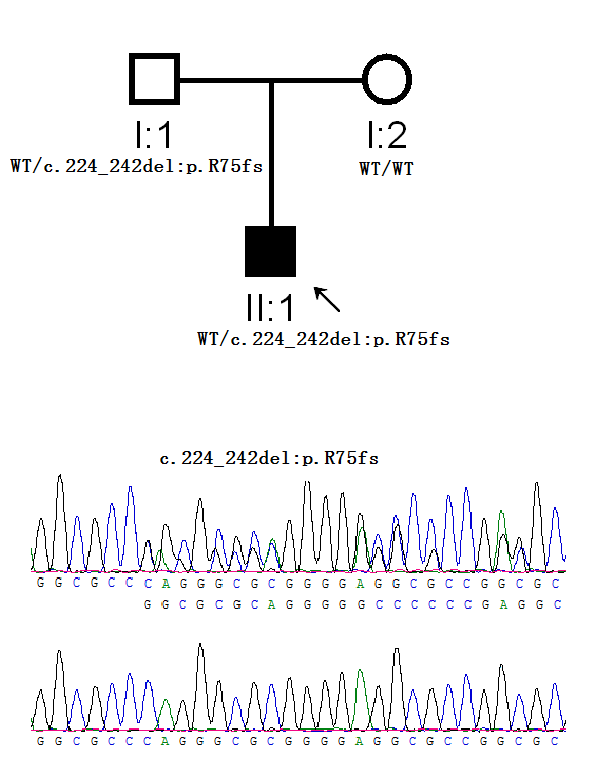


RS014
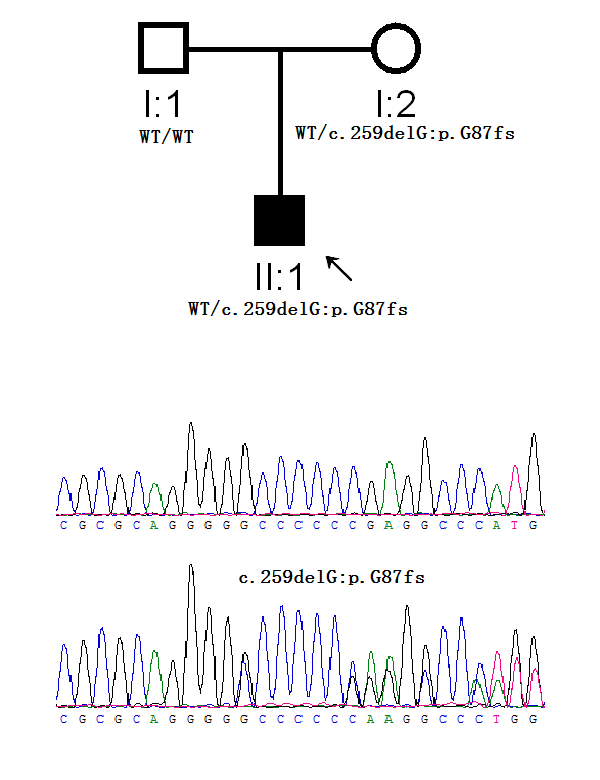


RJ037


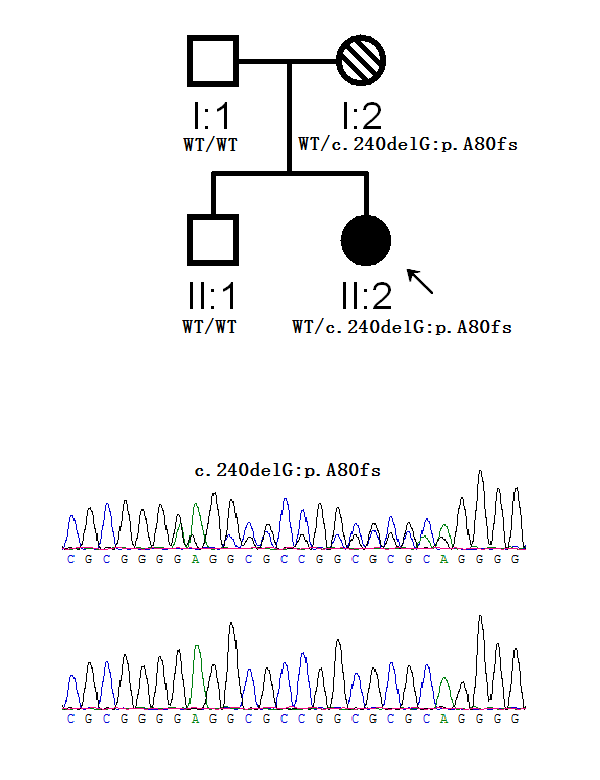


RJ027


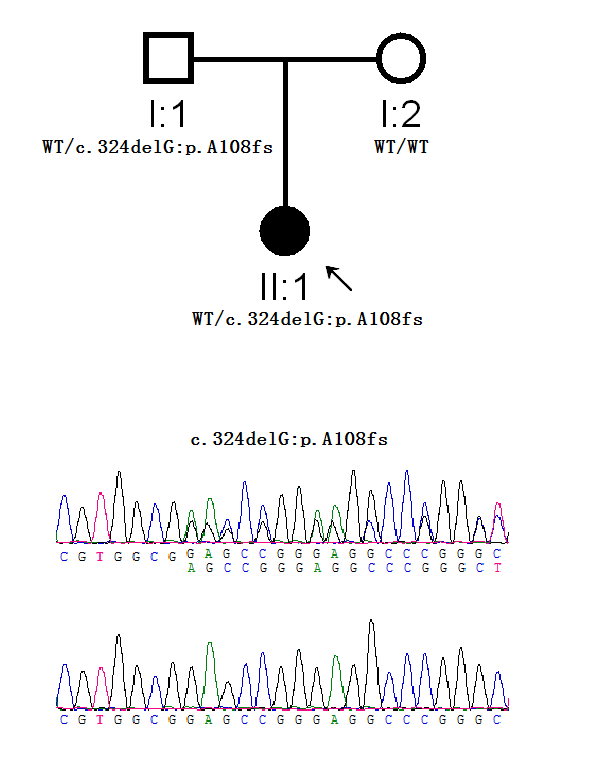


RJ004


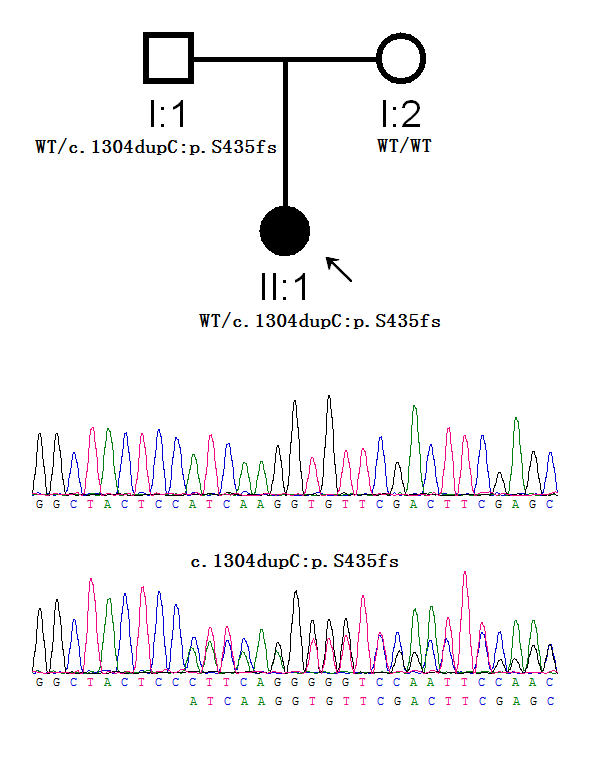


RJ002


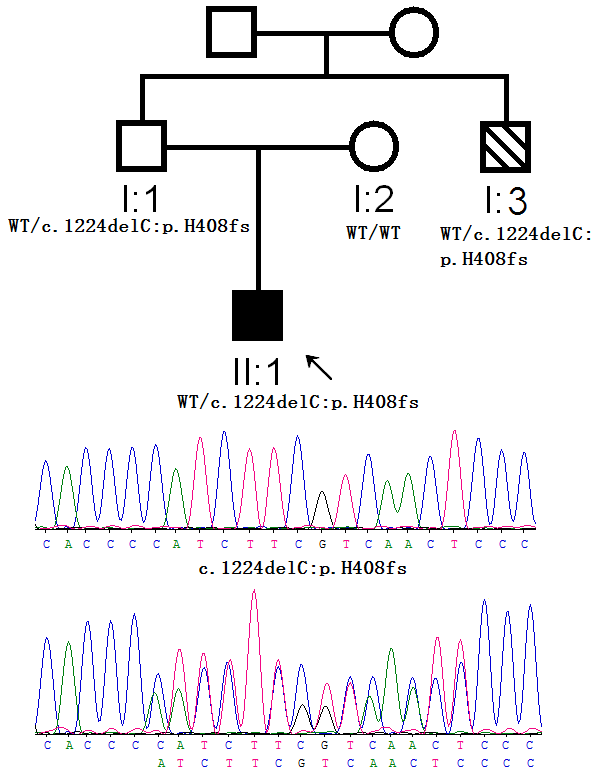


RCX001
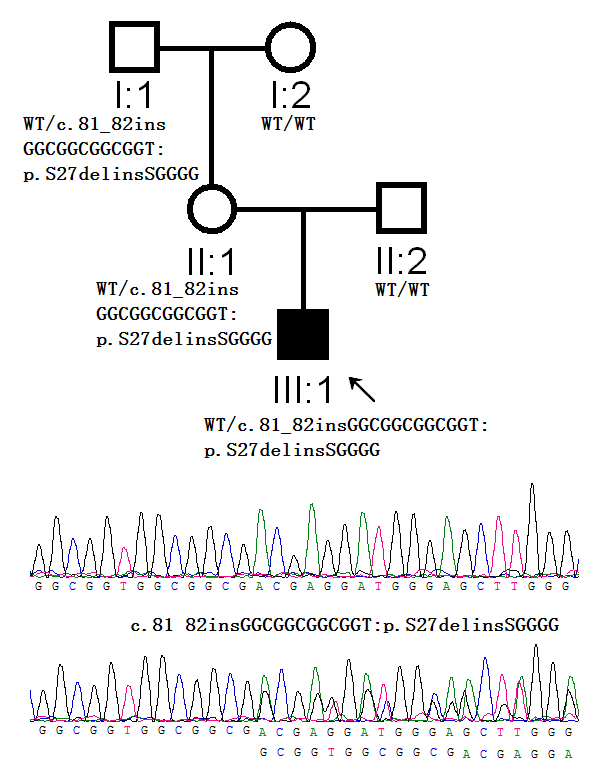


R118


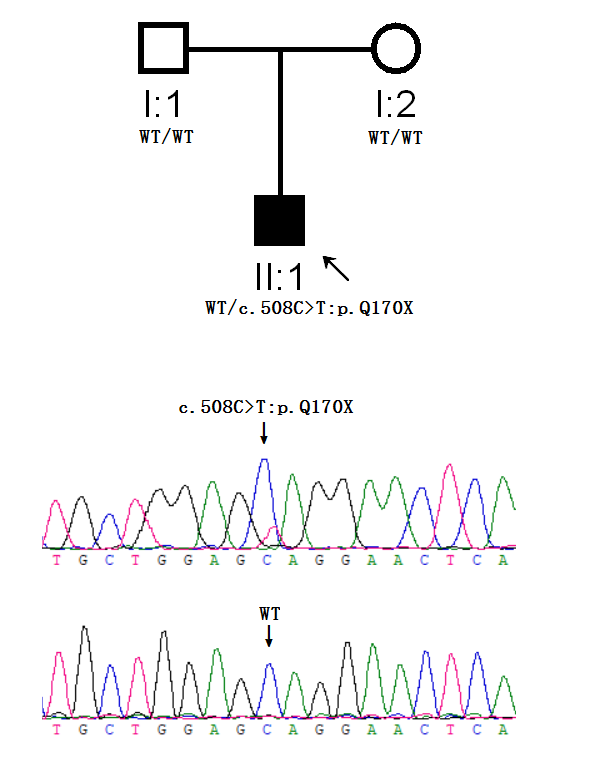


R088
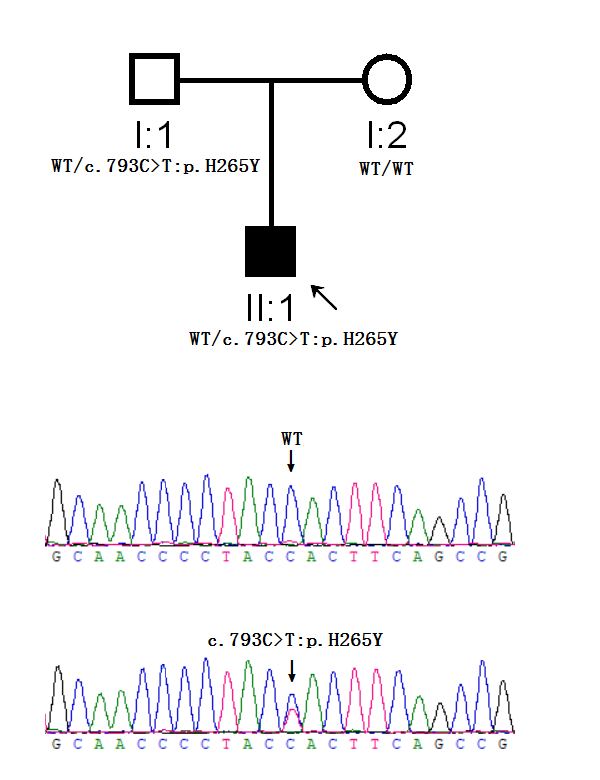


R078
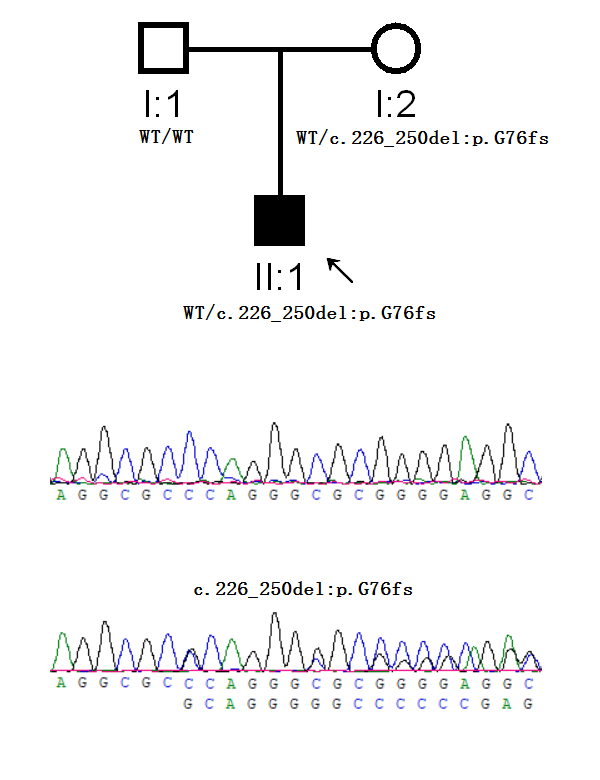


R076
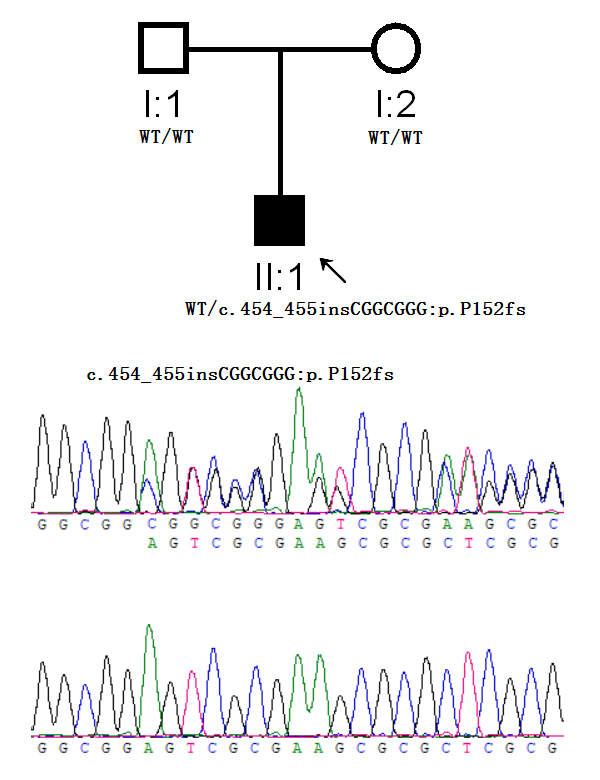


R074
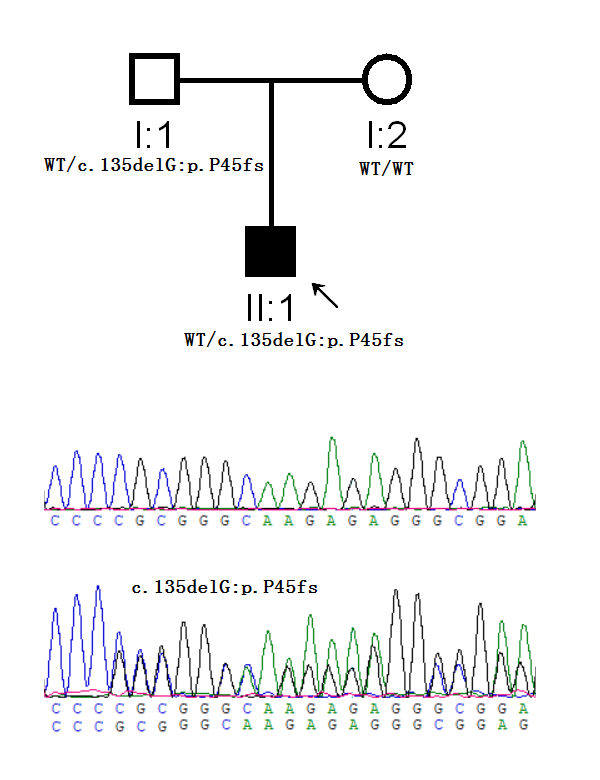


R073
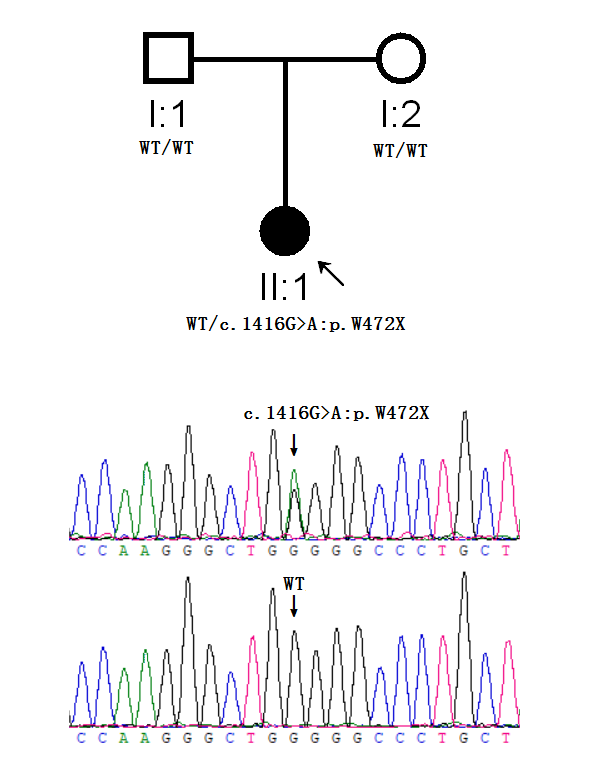


R052
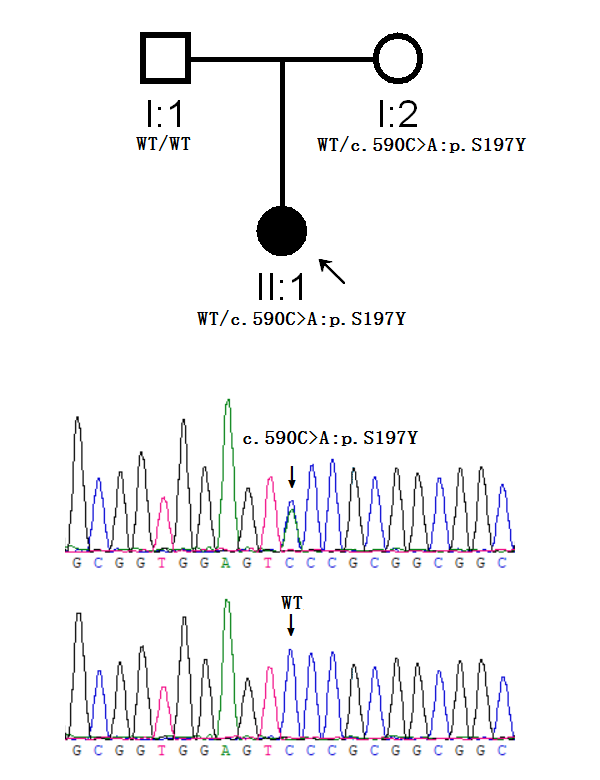


R041
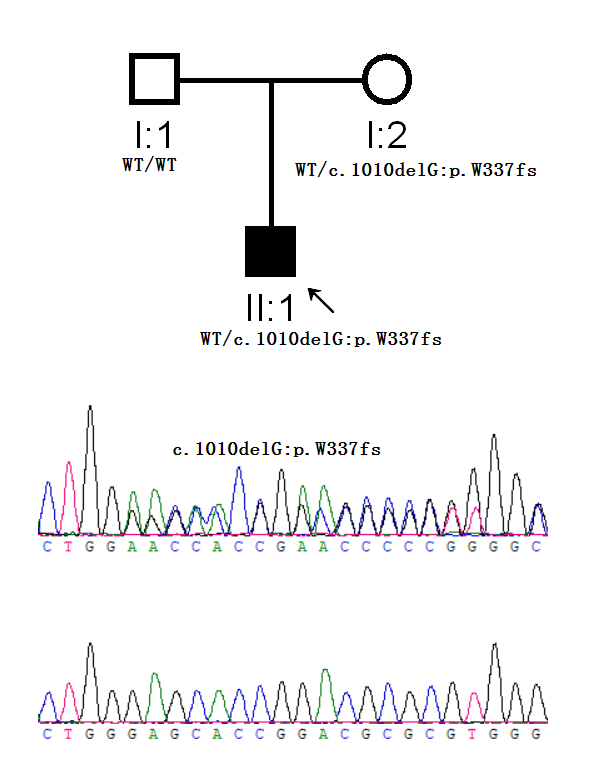


R035
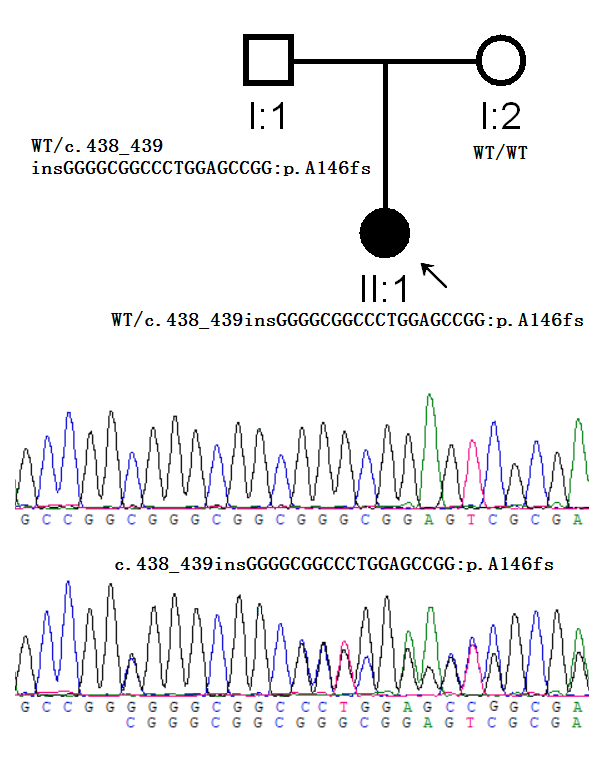


R028
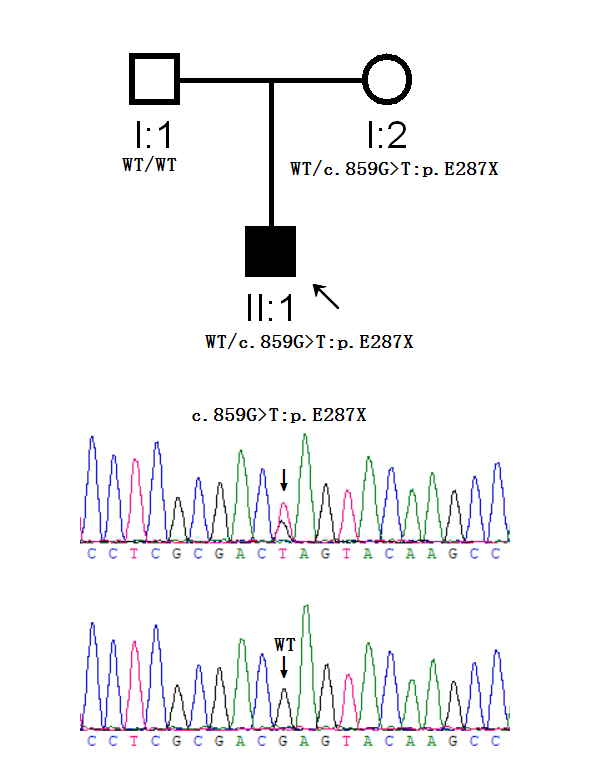


R026
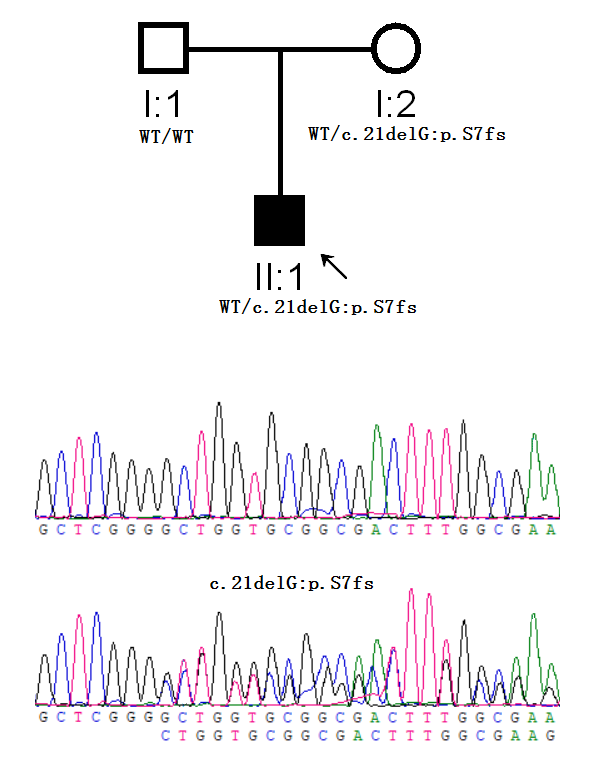


R005
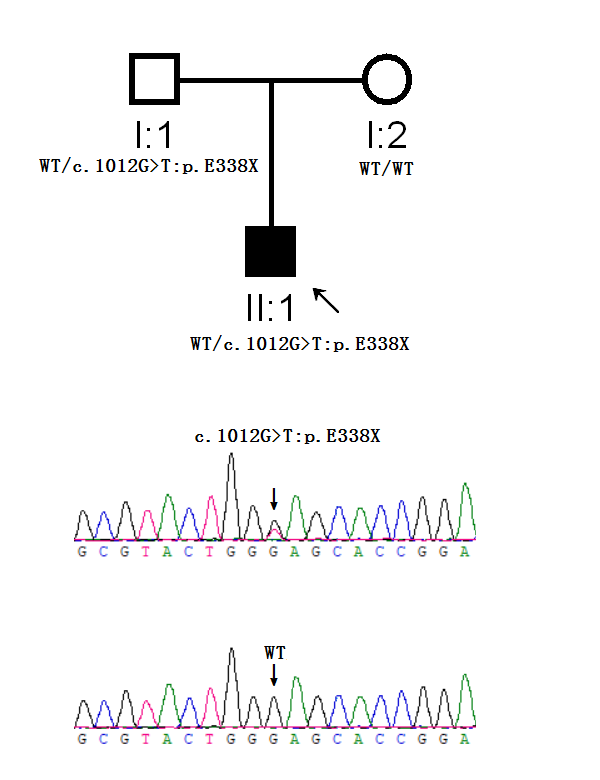


R004
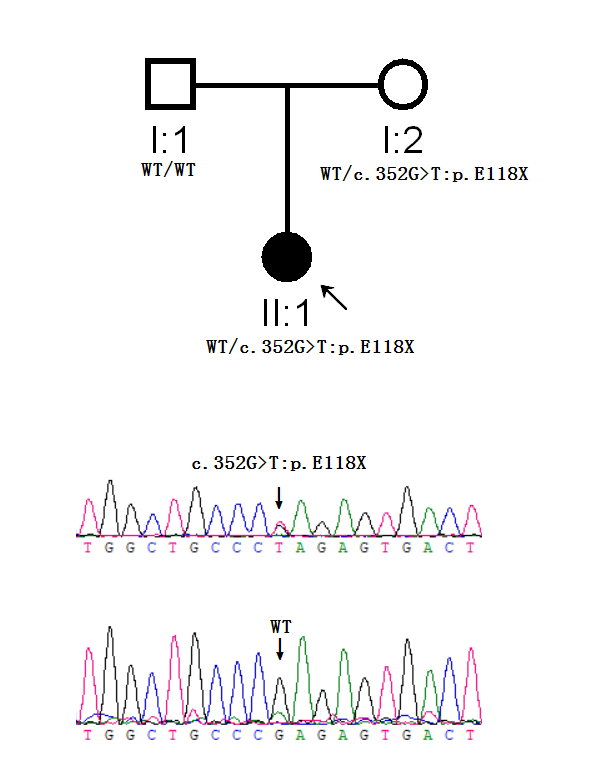


M4553
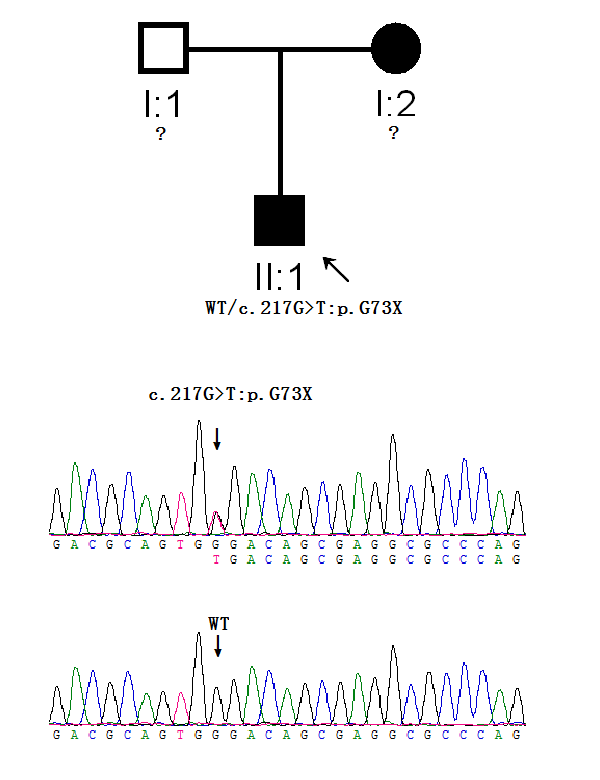


M4400
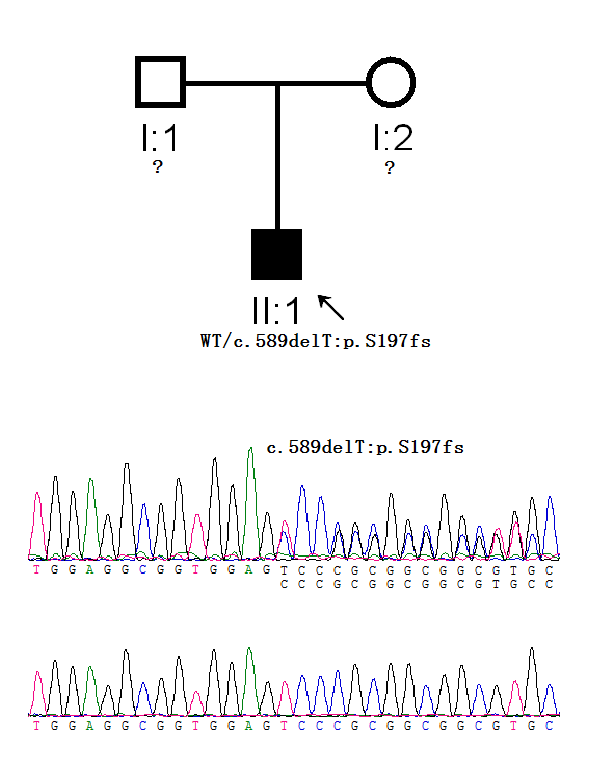


M4272
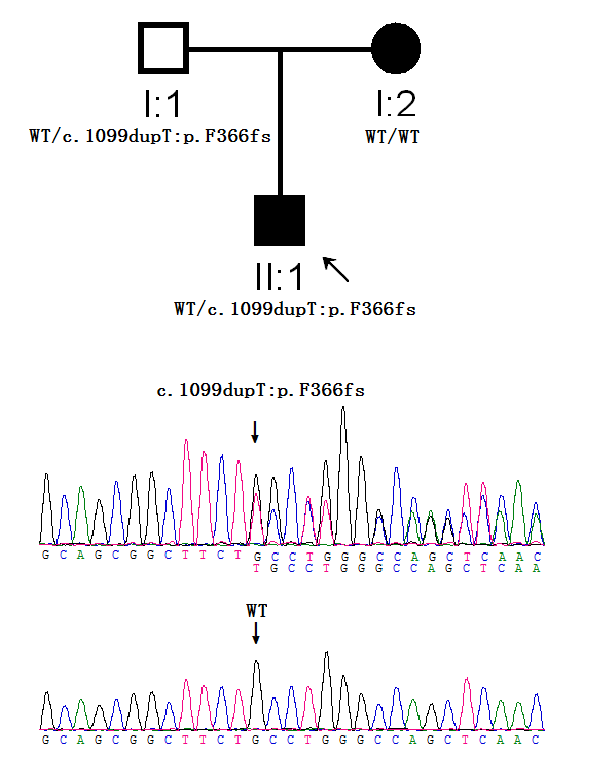


M3540
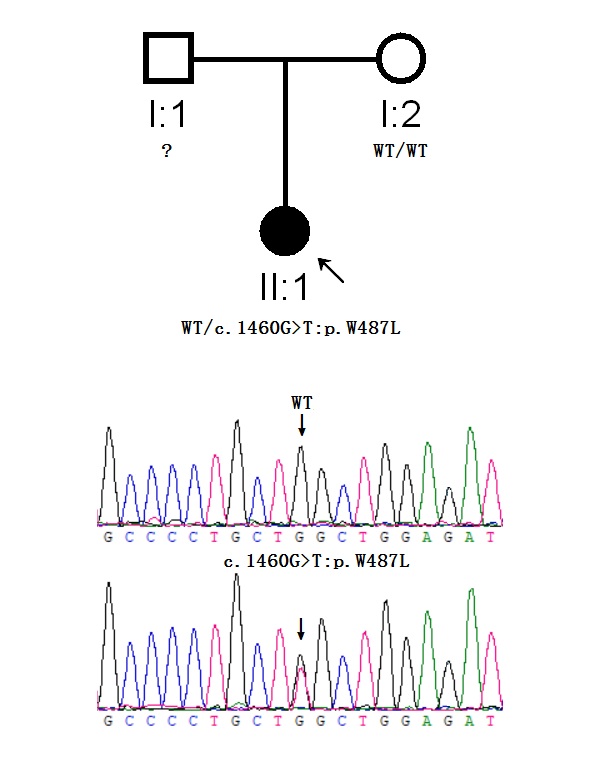


M3511
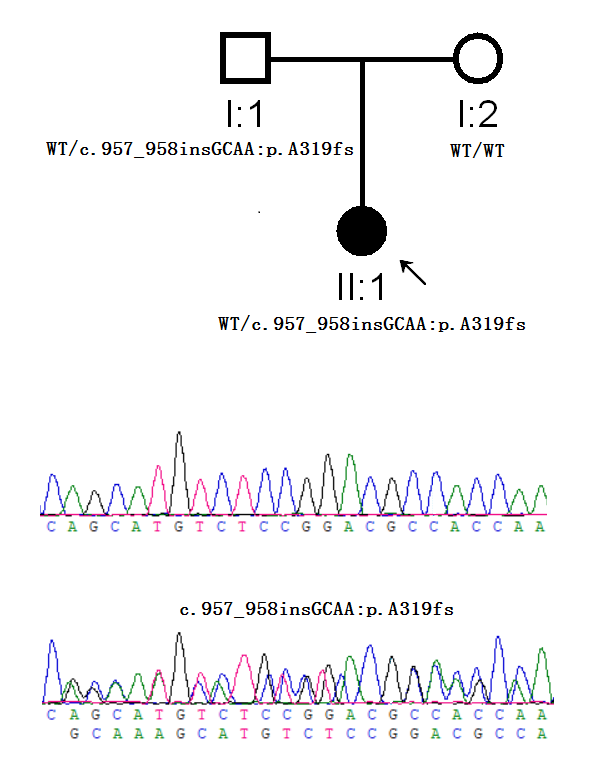


M3262
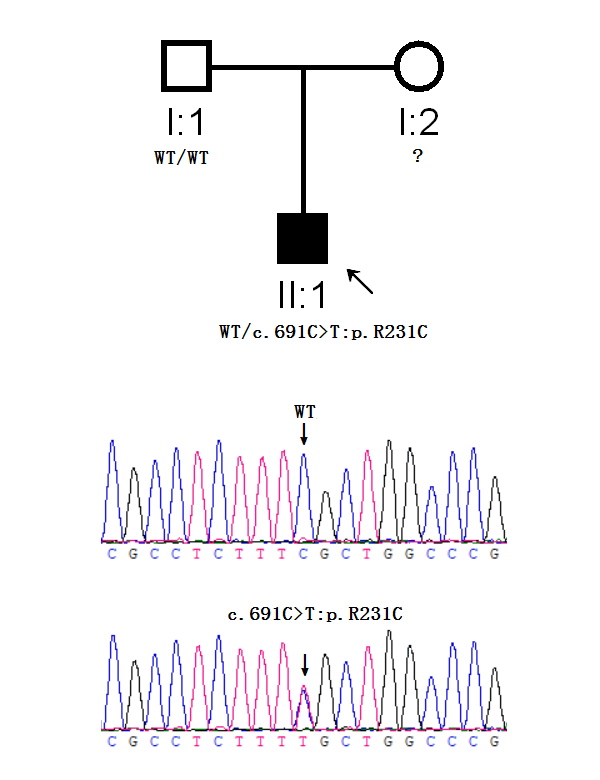


RS139
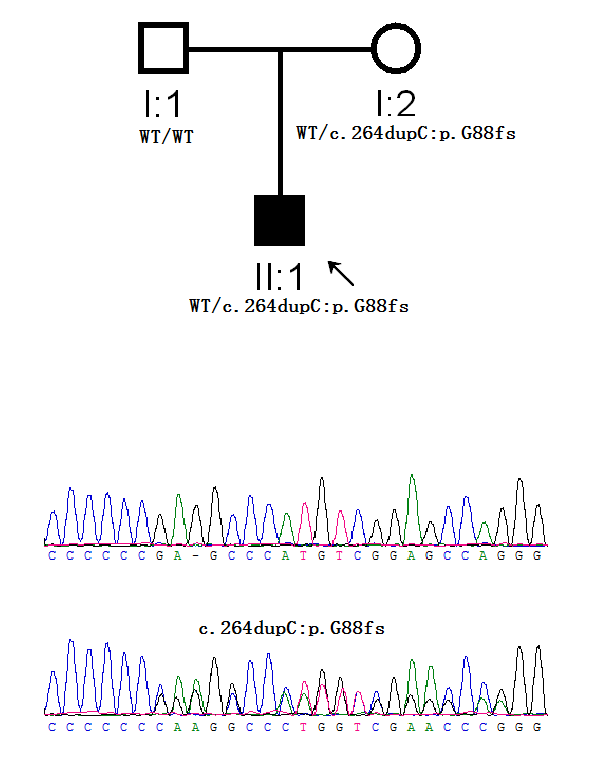

Supplement: Supplementary file 1 — Fig S1‐S2 [file MGG3-10-e1850-s001.docx]
