# Supplementary material for: A genotype and phenotype analysis of SMAD6 mutant patients with radioulnar synostosis
Source: Mol Genet Genomic Med. 2021 Dec 24;10(1):e1850. doi: 10.1002/mgg3.1850 (PMC8801148; doi:10.1002/mgg3.1850)
Supplement: Supplementary file 2 — Table S1‐S4 [file MGG3-10-e1850-s002.docx]

**Supplementary Table 1:** The cohort of patients with radioulnar synostois (RUS) studied in the present study.

Note: RUS Pedigree mean there are 2 or more RUS patients within a single family.

|  | **RUS Pedigree** | | |
| --- | --- | --- | --- |
|  | **Present study** | **Published (ref4)** | **All** |
| **All cohort** | 27 pedigrees | 11 pedigrees | 38 pedigrees |
| ***SMAD6* positive** | 13 pedigrees | 3 pedigrees | 16 pedigrees |
|  |  |  |  |
|  | **RUS Sporadic case** | | |
|  | **Present study** | **Published (ref4)** | **All** |
| **All cohort** | 268 cases | 125 cases | 393 cases |
| ***SMAD6* positive** | 37 cases | 24 cases | 61 cases |

**Supplementary Table 2:** Primers and PCR conditions used in the present study.

**Note 1:** for each PCR fragment products, the primers used in Sanger sequencing were forward primers, except for exon1, as the PCR products length for the exon1 was 980bp, to guarantee sequencing quality, another two primers (SMAD6-1-2R:ctccagcagcgtgtccag; SMAD6-1-3F:ctcttttcggagcgggac) were used. Note 2: only for positive sample, the related positions were replicated by Sanger sequencing using related reverse primers.

| **PCR Amplification Conditions** | | | | |
| --- | --- | --- | --- | --- |
|  | **Forward** | **Reverse** | **Annealing temp** | **Others** |
| Exon1 | CGCTGAGGGAACGGACCCCCGG | CCCGCAGCTGCGCCGACCCGCAGTG | 68°C | add 20% glycerin |
| Exon2 | ccatatctgtctgtgcccca | atgatgagccgcggaatttc | 60°C | - |
| Exon3 | caccacagtcagcactttcc | cattggcaaccttcccatcc | 60°C | - |
| Exon4-1 | gaaaccttacccagctccca | ggagttgacgaagatggggt | 66°C | - |
| Exon4-2 | ctgggccagctcaacctg | tgtgtctctgggcatcgg | 66°C | - |

**Supplementary Table 3:**  A descriptive table on the cohort characteristics (e.g. gender distribution, number of patients with bilateral, left or right RUS, average age, additional complications)

|  | **SMAD6+ pedigree patients:32** | **SMAD6+ sporadic patients:61** |
| --- | --- | --- |
| **Male** | 25 (78.13%) | 49 (80.33%) |
| **Female** | 7 (21.87%) | 12(19.67%) |
| **Bilateral RUS** | 22(68.75%) | 42 (68.85%) |
| **Left RUS** | 5 (15.63%) | 15 (24.59%) |
| **Right RUS** | 5 (15.63%) | 4 (6.56%) |
| **Average age (years)** | 5.1(0.75-22) | 5.2 (0.03-28.25) |
| **Additional complications** (Note: symptoms described  in Figure 3 were not shown in here) | None | 1 case has Pectus carinatum, asymmetric cranial development; 1 case has his mother had chromosome balanced translocation; 1 case has varicose testicular veins |

**Supplementary Table 4: In silico prediction of 12 *SMAD6* missense variants. Note:** The Eduardo Calpena model (ref10) used to predict the deleteriousness of *SMAD6* varaints was according to two criterias: DS>4 and the CADD predicted as damaging (CADD score>20).

| **Probands** | **Variant** | **SIFT=D** | **PolyPhen2=D** | **LRT=D** | **MutationTaster=D** | **GERP++≥5** | **PhyloP>1** | **DS score** | **CADD** |
| --- | --- | --- | --- | --- | --- | --- | --- | --- | --- |
| M3262 | SMAD6:c.691C>T | （+） | （+） | （+） | （+） | 3.96 | （+） | 5 | Damaging |
| M3540 | SMAD6:c.1460G>T | （+） | （+） | （+） | （+） | （+） | （+） | 6 | Damaging |
| R016 | SMAD6:c.38T>A | （+） | （+） | （+） | （+） | 3.79 | （+） | 5 | Damaging |
| R052 | SMAD6:c.590C>A | （+） | （+） | （+） | （+） | 2.67 | （+） | 5 | Damaging |
| R088 | SMAD6:c.793C>T | （+） | （+） | （+） | （+） | 4.23 | （+） | 5 | Damaging |
| R106 | SMAD6:c.1410G>C | （+） | （+） | （+） | （+） | （+） | （+） | 6 | Damaging |
| R107 | SMAD6:c.691C>A | （+） | （+） | （+） | （+） | 3.96 | （+） | 5 | Damaging |
| RS108 | SMAD6:c.791A>G | （+） | （+） | （+） | （+） | 4.23 | （+） | 5 | Damaging |
| RJ050 | SMAD6:c.793C>T | （+） | （+） | （+） | （+） | 4.23 | （+） | 5 | Damaging |
| RS134 | SMAD6:c.511G>A | （+） | （+） | （+） | （+） | 3.68 | （+） | 5 | Damaging |
| RS024 | SMAD6:c.572T>C | （+） | （+） | （+） | （+） | 3.68 | （+） | 5 | Damaging |
| RS077 | SMAD6:c.995G>T | （+） | （+） | （+） | （+） | （+） | （+） | 6 | Damaging |
